# Engineered π⋯π interactions favour supramolecular dimers X@[FeL_3_]_2_ (X = Cl, Br, I): solid state and solution structure†

**DOI:** 10.1039/d4sc01365d

**Published:** 2024-05-28

**Authors:** Arnau Risa, Leoní A. Barrios, Rosa Diego, Olivier Roubeau, Dmitry Y. Aleshin, Yulia Nelyubina, Valentin Novikov, Simon J. Teat, Jordi Ribas-Ariño, Guillem Aromí

**Affiliations:** a Departament de Química Inorgànica i Orgànica, Secció Química Inorgànica, Universitat de Barcelona Barcelona Spain leoni.barrios@ub.edu novikov@ub.edu aromi@ub.edu; b Institute of Nanoscience and Nanotechnology of the University of Barcelona (IN2UB) Barcelona Spain; c Instituto de Nanociencia y Materiales de Aragón (INMA), CSIC-Universidad de Zaragoza Zaragoza Spain; d Departamento de Física de la Materia Condensada, Universidad de Zaragoza Zaragoza Spain; e Kurnakov Institute of General and Inorganic Chemistry, Russian Academy of Sciences 119991 Moscow Russia; f Nesmeyanov Institute of Organoelement Compounds, Russian Academy of Sciences 119991 Moscow Russia; g Federal Research Center of Problems of Chemical Physics and Medicinal Chemistry, Russian Academy of Sciences Acad. Semenov Str. 1 Chernogolovka 142432 Russia; h Advanced Light Source, Berkeley Laboratory 1 Cyclotron Road Berkeley California 94720 USA; i Departament de Química Física, IQTCUB, Universitat de Barcelona Diagonal 645 08028 Barcelona Spain

## Abstract

Ditopic bis-pyrazolylpyridine ligands usually react with divalent metal ions (M^2+^) to produce dinuclear triple-stranded helicates [M_2_L_3_]^4+^ or, *via* π⋯π interactions, dimers of monoatomic complexes ([ML_3_]_2_)^4+^. The introduction of an additional benzene ring at each end of ligand L increases the number of aromatic contacts within the supramolecular aggregate by 40%, driving the self-recognition process in an irreversible manner. Consequently, the mixing of new bis-pyrazolylquinoline L2 with FeX_2_ salts leads to crystallization of the tripartite high-spin assemblies (X@[Fe(L2)_3_]_2_)^3+^ (X = Cl, Br or I). The aggregates exhibit exceptional stability, as confirmed by a combination of paramagnetic ^1^H NMR techniques, demonstrating their persistence in solution. Our investigations further reveal that the guests Br^−^ and I^−^ are retained inside the associate in solution but Cl^−^ is immediately released, resulting in the formation of the empty supramolecular dimer ([Fe(L2)_3_]_2_)^4+^.

## Abstract 2

The engineered supramolecular recognition of coordination complexes achieved by ligand design is shown in the solid state and in solution *via* X-ray diffraction and paramagnetic H-NMR spectroscopy.

## Graphical abstract

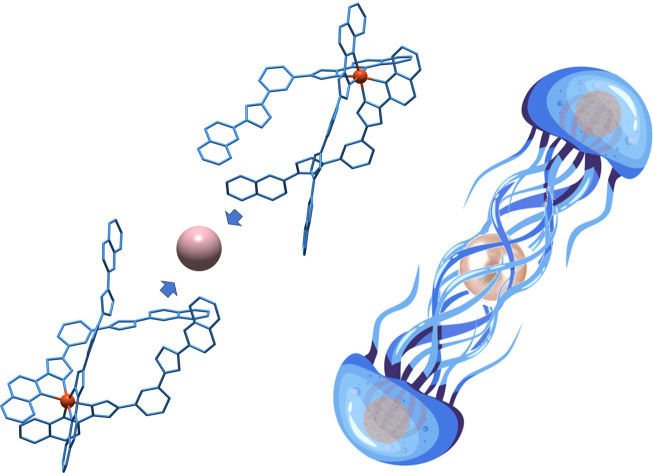

## Introduction

**Fig. 1:**
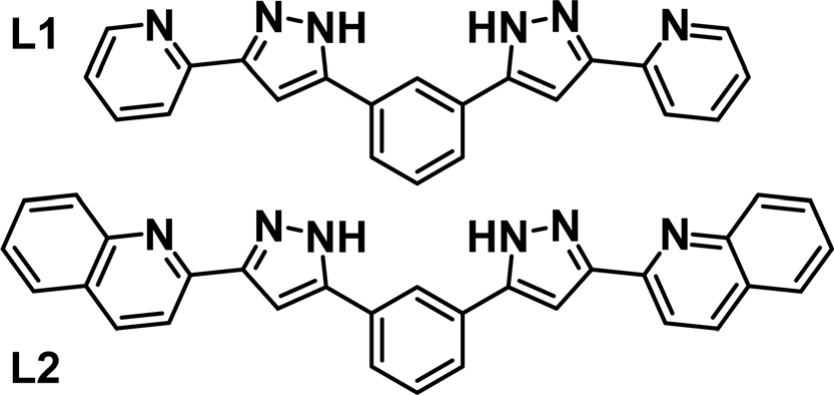
Ligands 1,3-bis-(1-(pyridin-2-yl)-pyrazol-3-yl)-benzene (L1), 1,3-bis(3-(quinolin-2-yl)-1*H*-pyrazol-5-yl)-benzene (L2).

Intermolecular interactions are at the heart of molecular recognition processes, driving the formation of supramolecular architectures.^1^ These assemblies are ubiquitous in nature and are of paramount importance for most biological processes.^2^ These principles have certainly provided inspiration for the design of artificial supramolecular assemblies^3^ and molecular machines.^4^ Their engineering requires a judicious design to put into action the adequate intermolecular interactions, which include for the most part hydrogen bonds or π⋯π, C–H⋯π, cation⋯π and anion⋯π contacts.^5–8^ The literature brings many examples of impressive supramolecular architectures achieved in this manner, ranging from discrete assemblies^9–13^ to polymeric supramolecular aggregates.^9,14–16^ An inclusive conception of the above also embraces the coordination bond as potentially labile interaction favoring self-sorting and stabilization of supramolecular architectures.^17,18^ Of these, one important category is that of metallohelicates with general composition [M_*x*_L_*y*_]^*m*^ (*m* = 0 or *n*+, M = metal ion, L = ligand strand),^19–21^ which often allow exploitation of their central cavity to accommodate guests *via* intermolecular interactions.^22–25^ We have been engaged for some years on the design of ligands, L (Fig. 1), featuring two pyrazolylpyridine moieties separated by an aromatic spacer and prone to generate G@[M_2_L_3_]^*m*^ (G = various guests) helical architectures.^26^ When M = Fe(ii), the coordination environment engendered by these ligands favours the observation of spin crossover (SCO) properties,^27^ as reported with other related ligand types.^28–32^ This has allowed us to explore the synergy between SCO and other functional properties, such as single ion magnetism,^33^ ligand photo-isomerization^34^ or electronic spin quantum coherence.^35^

During the course of these investigations, we discovered that ligand L1 (Fig. 1) can arrange in two different supramolecular organizations of similar energy when coordinated to Fe(ii) in the presence of Cl^−^ or Br^−^ anions (X^−^). These are either the expected ([Fe_2_(L1)_3_])^4+^ helicate cation encapsulating X^−^ in the central cavity,^27,36^*i.e.* (X@[Fe_2_(L1)_3_])^3+^, or a dimer of two [Fe(L1)_3_]^2+^ mononuclear complexes, interacting by interdigitation of the uncoordinated ends of ligands L1 while holding an X^−^ anion in between them, *i.e.* (X@[Fe(L1)_3_]_2_)^3+^.^37^ Both systems can be isolated in the solid state, by adjusting the conditions of the synthesis, while they can easily coexist in solution. The larger versatility of the dimeric assembly allows also trapping the anion I^−^, which does not fit into the cavity of the helicate. In addition to 12 strong hydrogen bonding interactions, the dimer of [Fe(L1)_3_]^2+^ species holds together thanks to three arrays of five π⋯π interactions each (a total of 15 such interactions).

We report here a chemical engineering method in order to obtain exclusively (X@[Fe(L2)_3_]_2_)^3+^ dimers while suppressing completely the formation of the corresponding helicate. The method consists of fusing an additional aromatic ring at each end of the bis-pyrazolyl ligand. The new ligand, L2 (Fig. 1), furnishes only the (X@[Fe(L2)_3_]_2_)^3+^ (X^−^ = Cl^−^, 1; Br^−^, 2; I^−^, 3) assemblies, which, predictably, exhibit a total of six additional π⋯π contacts in comparison with the analogues with L1. A full ^1^H NMR analysis shows that the dimers stay together in MeCN solution and proves that they remain the sole species in this medium. The tendency for dimerization was also supported by DFT calculations.

## Results and discussion

### Synthesis

Ligand L2 was prepared in an analogous way to L1,^27^ by carrying out a Claisen condensation reaction between methyl quinoline-2-carboxylate and 1,3-diacetophenone to obtain a bis-β-diketone precursor (see ESI†). Cyclisation of the 1,3-dicarbonyl moieties of this intermediate (Fig. S1†) into pyrazolyl rings was achieved by reaction with hydrazine (Fig. S2†). Suspensions of L2 in acetone were mixed with solutions in the same solvent of FeX_2_ (X = Cl^−^, Br^−^, I^−^), followed by the addition of ^*n*^Bu_4_NPF_6_. Upon diffusion of ether, the filtrates of these reactions produced, respectively, crystals of X@[Fe(L2)_3_]_2_(PF_6_)_3_ (X = Cl^−^, 1; Br^−^, 2) and I@[Fe(L2)_3_]_2_(I_3_)_0.39_(PF_6_)_2.61_ (3) that were amenable to single crystal X-ray diffraction (SCXRD). The bulk microanalysis results are consistent with their formulation in all cases (ESI†).

### Molecular structures

**Fig. 2:**
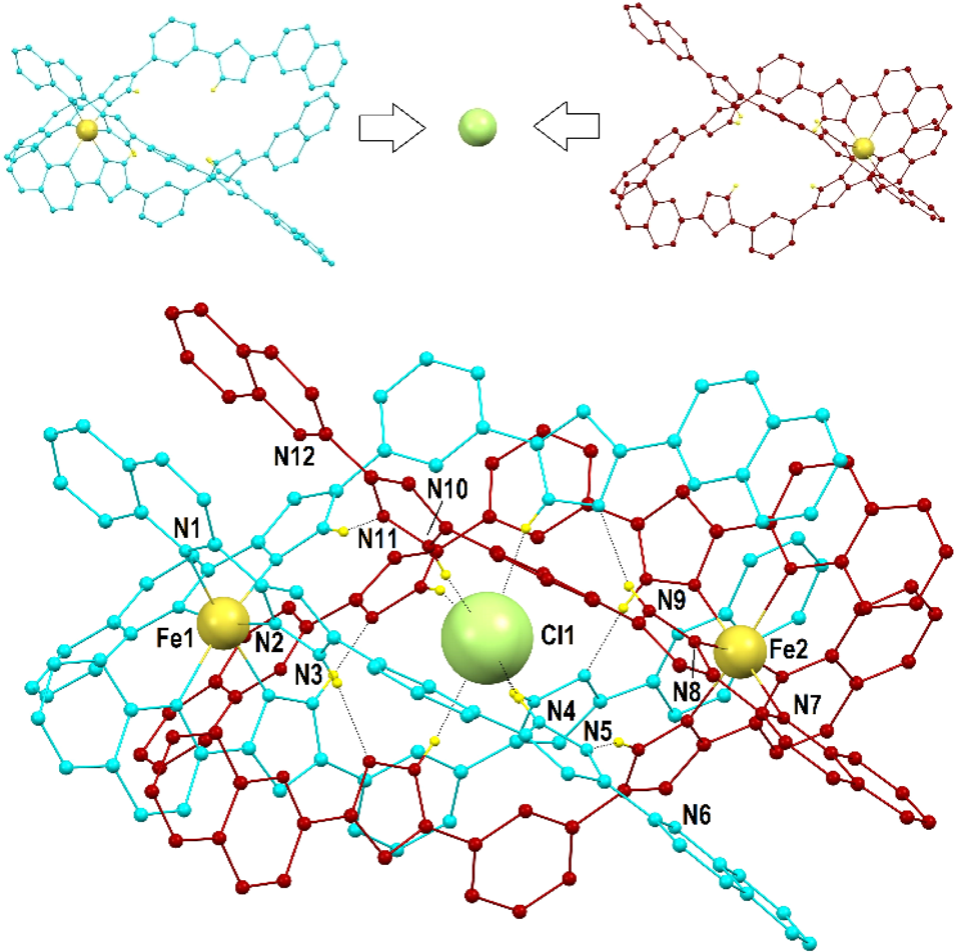
(Top) representation of both [Fe(L2)_3_]^2+^ monomers in 1 emphasizing the way in which they come together, encapsulating a Cl^−^ ion in between them. (Bottom) molecular representation of the (Cl@[Fe(L2)_3_]_2_)^3+^ assembly of 1. The ligands of each [Fe(L2)_3_]^2+^ unit are shown in different colours (burgundy and turquoise, respectively). Unique heteroatoms are labelled. Small yellow balls are H (only H atoms from N–H groups are shown), and dashed lines are H-bonds.

**Fig. 3:**
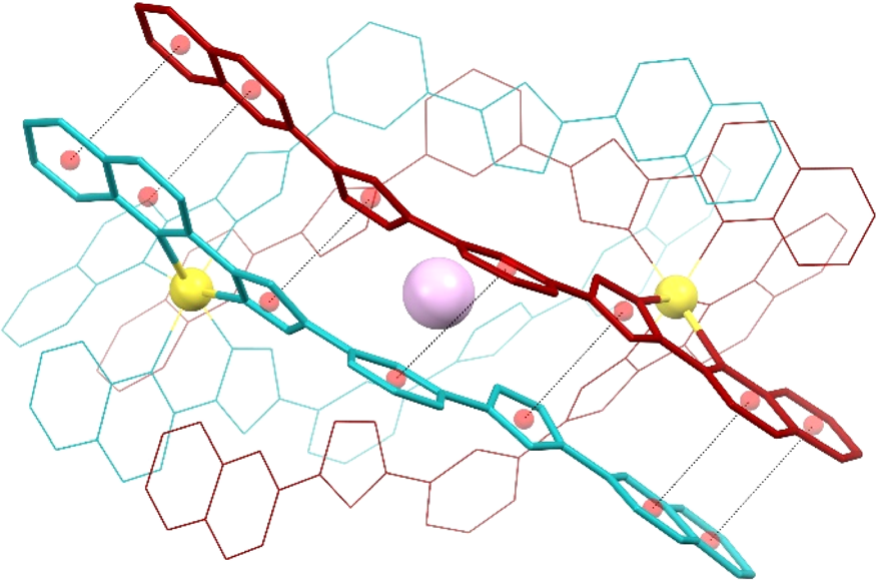
Representation of the supramolecular cation (I@[Fe(L2)_3_]_2_)^3+^ of 3 (also representing 1 and 2) emphasizing in “capped stick” style one of the three sets of seven π⋯π interactions that contribute to holding the assembly together (for the appropriate inter-centroid distances and interplane angles, see Table S4†). The ligands of each [Fe(L2)_3_]^2+^ unit are shown in different colours (burgundy and turquoise, respectively). Central purple ball is I, yellow balls are Fe, and red balls are centroids of the aromatic rings involved in the stacking interactions (shown as dashed lines). H atoms are not shown.

Compounds 1 to 3 were characterized by SCXRD (Fig. 2, 3 and S3–S6, and Tables S1–S4†) at 100 K. They are dimeric supramolecular assemblies X@[Fe(L2)_3_]_2_(PF_6_)_3_ (X = Cl^−^, 1; Br^−^, 2) and I@[Fe(L2)_3_]_2_(I_3_)_0.39_(PF_6_)_2.61_ (3) that crystallize in the cubic lattice of the Sohncke space group *P*2_1_3. Their asymmetric unit contains one third of their formula composition with two Fe centres and one halide anion located on a 3-fold symmetry axis. The assemblies consist of two [Fe(L2)_3_]^2+^ complexes, each featuring a pseudo-octahedral Fe^2+^ centre chelated by three L2 ligands through pyrazolyl/quinoline pockets. The average Fe–N bond distances (in the Fe1/Fe2 format) of 2.231(8)/2.221(8), 2.232(13)/2.231(15) and 2.233(6)/2.225(8) Å (for 1, 2 and 3 respectively) indicate that all the Fe centres are in the high-spin (HS) state.^38^

Both complexes dimerize through the interdigitation of the non-coordinated arms of ligands L2, assisted by the encapsulation of one halide anion X^−^ (Cl^−^, 1; Br^−^, 2; I^−^, 3; Fig. 2). The dimerization occurs through an extensive ensemble of intermolecular interactions. Those include six N–H⋯X hydrogen bonds between all the non-coordinated pyrazolyl rings and the central halide ion (Fig. 2, Table S3†). The average N⋯X distances, 3.358(7), 3.414(12) and 3.522(6) Å in 1, 2 and 3 respectively, are sensitive to the nature and size of the central X^−^ guest of the assembly. If corrected for the difference in the ionic radii of Cl^−^, Br^−^ and I^−^ (1.81, 1.96 and 2.16 Å, resulting in separations of 1.55, 1.45 and 1.36 Å respectively), they hint at the strengthening of the N–H⋯X hydrogen bonds from 1 to 3, which mirrors the counterintuitive decrease in the Fe⋯Fe distances from 12.147(2) to 11.957(2) Å (Table S2†) in going from the smaller-sized chloride anion to the bigger-sized iodide anion. Six other N–H⋯N hydrogen bonds occur between the coordinated pyrazolyl rings (acting as the H-donor) of the ligands of each complex and the non-coordinated pyrazolyl rings (acting as the H-acceptor) from the complex in front (Fig. 2, Table S3†). As gauged by the average N⋯N distances, 2.910(11), 2.906(19) and 2.943(9) Å in 1, 2 and 3, respectively, these are very similar among the three compounds, only slightly weaker in 3. The most numerous interactions holding the two complexes together are, however, parallel-displaced stacking interactions between aromatic rings of the ligands L2 interacting pairwise. The resulting three sets of seven unique contacts (Fig. 3), which may contribute *ca.* 4–5 kcal mol^−1^ each,^39^ are very robust. The average inter-centroid distances and interplane angles in 1–3 (Table S4†) differ only by 0.02 Å and 0.27°, respectively, confirming their key role in the stabilization of the supramolecular dimer in 1–3. Another piece of experimental evidence for the key role of quinoline moieties for the stacking interactions comes from the previously reported analogous iron(ii) complexes of the ligand L1 (with pyridine instead of quinoline).^37^ They produce similar supramolecular dimers with less efficient stacking interactions, due to the rotation of the non-coordinating pyridine ring. This could in part explain the formation of the other arrangement ([Fe_2_(L1)_3_]^4+^ helicates) not observed with the ligand L2.

The (X@[Fe(L2)_3_]_2_)^3+^ supramolecular dimers are chiral (Fig. S5†); the local chirality of the tris-chelate six-coordination on the metal propagates into the structural chirality of the [Fe(L2)_3_]^2+^ complex. Complexes of the same handedness then recognize each other. Each single crystal contains assemblies of only one chirality; therefore, optical resolution occurs during crystallization, as follows from near-zero values of the Flack parameters (see Table S1†). Indeed, many crystals have been measured by SCXRD and both handedness (*Δ* and *Λ*) have been characterized (*i.e.* either one or the other) irrespective of the compound.

Variable temperature bulk magnetic susceptibility measurements confirm in the 5 to 300 K temperature range the spin-state observed crystallographically at 100 K for the Fe^2+^ centres in 1, 2 and 3. The HS state is maintained at all temperatures, down to 4 K (Fig. S7†).

### Paramagnetic NMR study

**Fig. 4:**
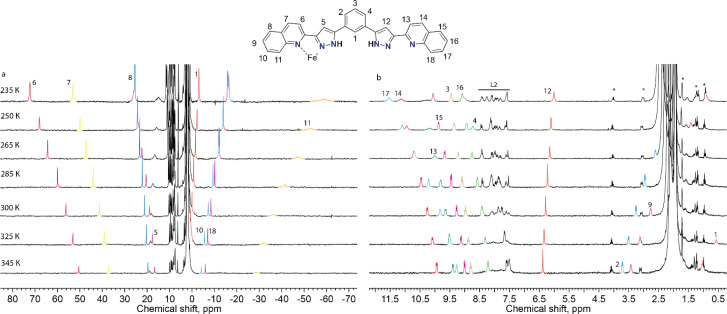
Paramagnetic (a) and diamagnetic (b) regions of the ^1^H NMR spectrum of (Br@[Fe(L2)_3_]_2_)^3+^ (CD_3_CN, 300 MHz) at various temperatures. The color coding designates the signals of the same proton at different temperatures to guide the eye. The signals corresponding to the free ligand are labelled as L2. Due to the low solubility of the complex in acetonitrile, the signals of small admixtures (such as grease, ethyl acetate and ^13^C satellites of the residual solvent signals) appear magnified; they are designated by asterisks.

To probe the integrity of these supramolecular assemblies devoid of crystal packing effects, we used solution-state paramagnetic ^1^H-NMR spectroscopy. It is known that the analysis of NMR spectra of paramagnetic coordination compounds is particularly challenging, but may be very informative; the chemical shifts caused by paramagnetic ions often help elucidating the structure of the molecules in solution^40^ and even the peculiarities of their dynamics.^36^ Therefore, we chose this method to determine if the supramolecular assembly of two unsymmetric [Fe(L2)_3_]^2+^ complexes survives in solution. NMR spectra of the chlorine-, bromine- and iodine-containing compounds demonstrated very similar behaviour. Therefore, only one of them, (Br@[Fe(L2)_3_]_2_)^3+^, is discussed here in detail (Fig. 4). The spectra and their assignments for the other two compounds are given in the ESI (Fig. S8 and S9, Table S5†).

For 2, the ^1^H NMR spectrum at room temperature in acetonitrile-*d*_3_ features a set of strongly shifted (paramagnetic) signals together with several conventional (diamagnetic) peaks. As ligand L2 has two-fold symmetry, only ten signals are expected for the 18 non-exchangeable protons of a free ligand or of a hypothetical [Fe_2_(L2)_3_]^2+^ helical complex (expected to display idealized *D*_3_ symmetry). An asymmetric complex, however, would produce an individual signal for each ^1^H nucleus of L2. In the ^1^H NMR spectrum, there are eight paramagnetic signals (four in a weak field and four in a strong field region of the spectrum, Fig. 4) and many diamagnetic signals, including those from the small admixture of the free ligand. In contrast to the signals of the free ligand, all signals of the species (Br@[Fe(L2)_3_]_2_)^3+^, from 2, show temperature-dependent paramagnetic shifts (most very large) that follow the Curie law^41^ (Fig. 4), implying that only the HS state of the complex is populated in the probed temperature range, as also expected from magnetic susceptibility measurements in the solid state.^42^ Note that we did not observe any significant changes in the number of signals or their linewidths, suggesting the absence of any chemical exchange between different species in solution, including the dissociation of the complexes or interactions with the traces of the free ligand present.

While the asymmetry of the complex directly follows from the observed pattern of the paramagnetic shifts, the same pattern could arise from either the dimeric assembly observed in the solid state or from a hypothetical monomeric [Fe(L2)_3_]^2+^ species. As these two structures are different in size, they can be easily singled out by diffusion-ordered NMR spectroscopy (DOSY), which furnishes a diffusion coefficient of a compound in solution allowing an estimation of its size. With a small amount of the free ligand L2 as an internal standard, the diffusion coefficients for the assembly (Br@[Fe(L2)_3_]_2_)^3+^ and for the free ligand were estimated as 5.89 × 10^−6^ and 9.66 × 10^−6^ cm^2^ s^−1^, respectively (Fig. S10†). These coefficients are related to the hydrodynamic radii *via* the Stokes–Einstein equation,^43^ so that the third power of their ratio, *i.e.* (9.66/5.89)^3^ = 4.4, thus corresponds to the ratio of the hydrodynamic volumes of the two species. The resulting value of 4.4 is significantly larger than 3, as expected approximately for a hypothetical monomeric species [Fe(L2)_3_]^2+^. This strongly indicates the presence of the known dimer (Br@[Fe(L2)_3_]_2_)^3+^ in solution. Note that while the signal of the residual free ligand appears in the spectra of all complexes, its integration shows that less than 10% of the totality of L2 present in solution is in its free form (Fig. S11†). NMR 2D EXSY experiments were performed to assess the possibility of slow exchange between the ligand in its bound and free states but detected no exchange cross-peaks (Fig. S12†). Thus, while it is possible that a small amount of the complex dissociates upon dissolution, the formed free ligand either is not in exchange with the original compound or exchange is too slow to be observed by available methods.

**Fig. 5:**
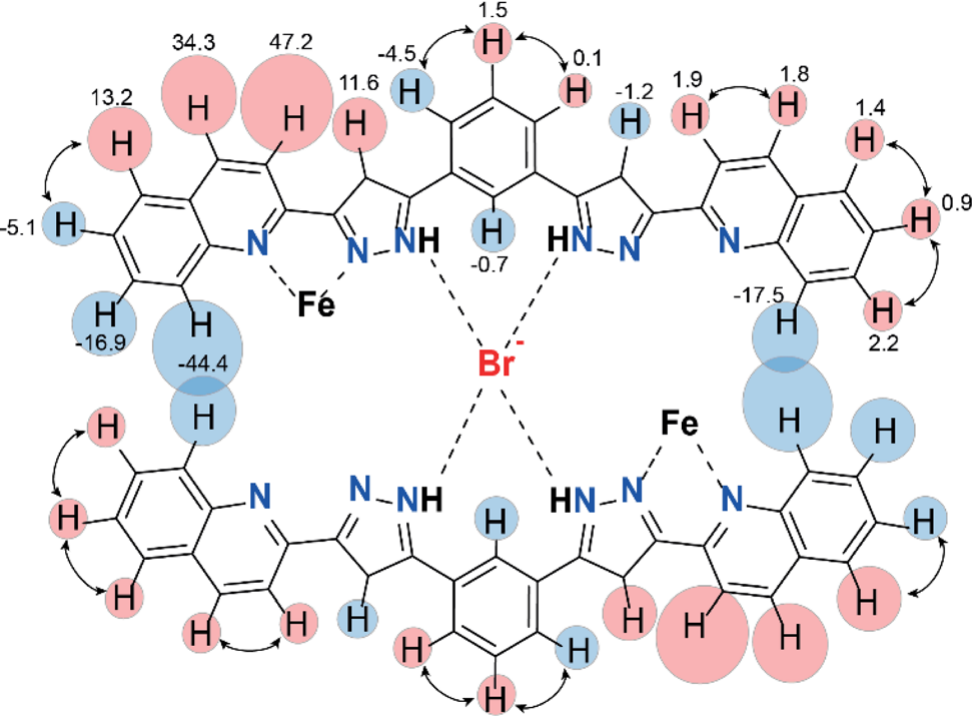
Paramagnetic shifts for the protons of the assembly (Br@[Fe(L2)_3_]_2_)^3+^ obtained as a difference between experimentally observed chemical shifts of the complex at 235 K and those of the free ligand at RT. Only two ligands out of six are shown for clarity. Red and blue circles show downfield and upfield paramagnetic shifts, respectively. The size of the circles correlates with the absolute value of the shift. The arrows connect the protons for which the correlations in COSY spectra are observed.

Further evidence for the dimeric nature of compound 2 in solution comes from a more detailed analysis of the observed paramagnetic shifts. For an unsymmetrical monomeric complex [Fe(L2)_3_]^2+^, one would expect the signals of the ligand fragments close to the coordinated iron to be strongly paramagnetically shifted while protons increasingly distant from the metal gradually appearing closer to the diamagnetic range of chemical shifts. Likewise, the dimer (Br@[Fe(L2)_3_]_2_)^3+^ is predicted to have the “diamagnetic” part of the ligands of one complex close to the iron(ii) ion of the other complex, therefore, also experiencing a paramagnetic shift. As the magnetic anisotropy of the iron(ii) ions in a trigonal environment is generally low,^44^ the paramagnetic shifts caused by this ion are mainly due to through-bond contact interactions. Nevertheless, non-zero pseudo contact through-space interactions with Fe(ii) can cause the shift of protons lying nearby, despite belonging to a different complex. This is consistent with the pattern of signals observed in the NMR spectra of 2 (Fig. 5).

Thus, the combined evidence from diffusion and paramagnetic NMR data, along with the absence of any additional signals across the explored temperature range of over 100 K, confidently supports the presence of only dimeric compounds in the acetonitrile solution.

The assignment of the signals is assisted by 2D-NMR spectroscopy and comparison with the spectra of the previously reported dimeric assemblies (X@[Fe(L1)_3_]_2_)^3+^.^37^ Despite the severe broadening of some signals by the paramagnetic influence on their relaxation time, important correlations in the homonuclear COSY spectrum were observed (Fig. S13 and S14†). The correlations between protons 2, 3 and 4 of the bridging phenylene moiety (Fig. 5) indicate that the diamagnetic and paramagnetic signals of the spectra correspond to protons belonging to the same molecule, thereby confirming the elusive unsymmetric geometry of the individual [Fe(L2)_3_]^2+^ cores. For comparison, the COSY spectrum of the free ligand L2 was also determined (Fig. S15†).

**Fig. 6:**
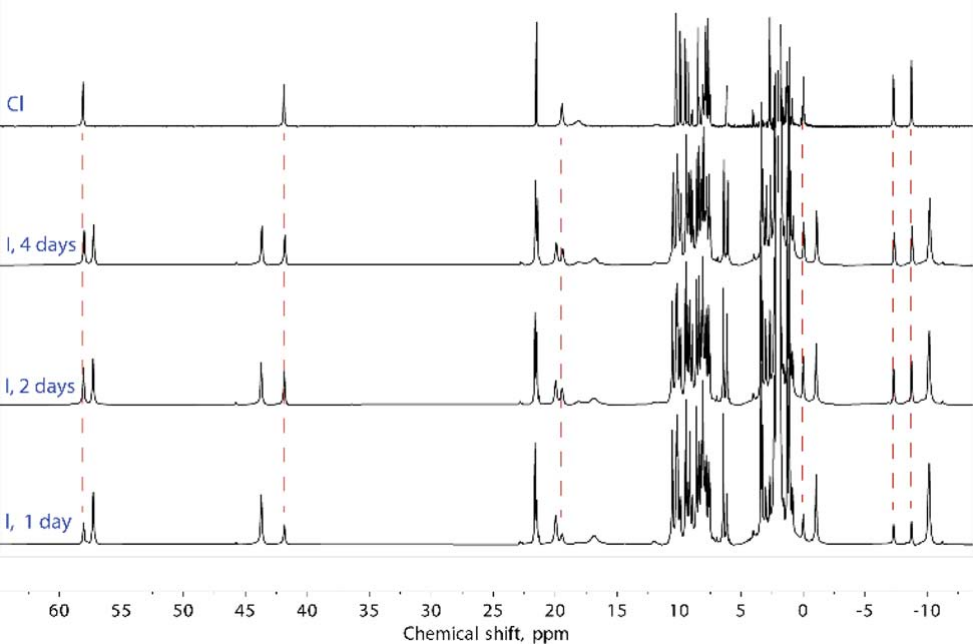
^1^H NMR spectra of (I@[Fe(L2)_3_]_2_)^3+^, from 3, and ([Fe(L2)_3_]_2_)^4+^, from 1. Dashed lines relate the signals that appear from compound (3) over the course of several days at room temperature with the signals of compound (1).

Solutions of compounds 1 and 3 (containing (Cl@[Fe(L2)_3_]_2_)^3+^ and (I@[Fe(L2)_3_]_2_)^3+^, respectively) produced very similar spectra, confirming that these compounds also retain the dimeric arrangement in solution (Fig. 6, S8 and S9†). The temperature dependence of the paramagnetic shifts (Fig. 4 and S9†) also follows the Curie law, thereby proving the metals to be HS in the studied temperature range. However, it was observed that the assembly (I@[Fe(L2)_3_]_2_)^3+^, from 3, yields over time another species showing a set of paramagnetic signals with the same unsymmetric pattern observed for all the other compounds studied. These new signals match exactly those observed for the assembly supposed to be (Cl@[Fe(L2)_3_]_2_)^3+^ recorded for 1. In the absence of any chloride ions in the solution of (I@[Fe(L2)_3_]_2_)^3+^, a halogen-exchange reaction is not possible, indicating that the only possible transformation of 3 is that part of the assembly (I@[Fe(L2)_3_]_2_)^3+^ loses the encapsulated iodide ion, becoming an “empty” ([Fe(L2)_3_]_2_)^4+^ aggregate. Consequently, the species formed upon dissolution of 1 (featuring (Cl@[Fe(L2)_3_]_2_)^3+^ aggregates in the solid state) also must consist of the aggregate ([Fe(L2)_3_]_2_)^4+^.

**Fig. 7:**
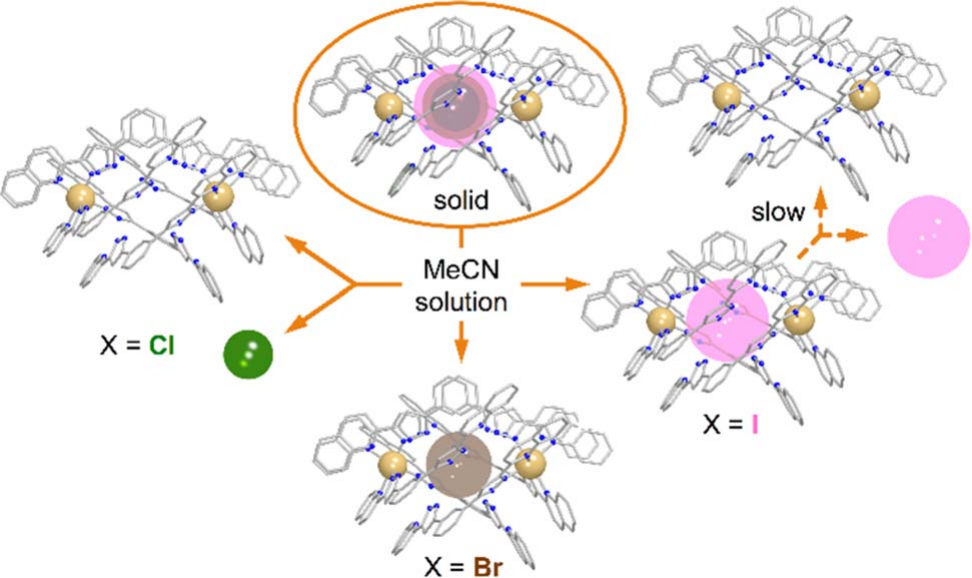
Schematic representation of the stability of the (X@[Fe(L2)_3_]_2_)^3+^ assemblies in the solid state and in solution as a function of the identity of X^−^ (Cl^−^, Br^−^ or I^−^).

To rule out the possibility of the formation of monomeric species, we performed DOSY ^1^H NMR for the solution of 3. The results provided strong evidence for the formation of the “empty” ([Fe(L2)_3_]_2_)^4+^ aggregate: the diffusion coefficients of the (I@[Fe(L2)_3_]_2_)^2+^ species and the newly formed compound were very similar, with less than a 10% difference (Fig. S16†). The species (I@[Fe(L2)_3_]_2_)^2+^ exhibits a slightly smaller diffusion coefficient, indicating only a marginally larger molecular size. This confirms that the dissolution of 1 produces gradually the aggregate ([Fe(L2)_3_]_2_)^4+^. From these observations it follows that, while dimetallic [Fe_2_(L1)_3_]^4+^ helicates are known to tightly encapsulate Cl^−^ and Br^−^ (but not I^−^) ions inside their three-dimensional cavity, the dimeric assembly ([Fe(L2)_3_]_2_)^4+^ in solution only retains the bromide anion strongly bound. In turn, the bulkier iodide anion slowly leaches into the solution upon dissolution of 3 whereas the smaller chloride anion is lost completely into the solution immediately when 1 is dissolved (Fig. 7).

**Fig. 8:**
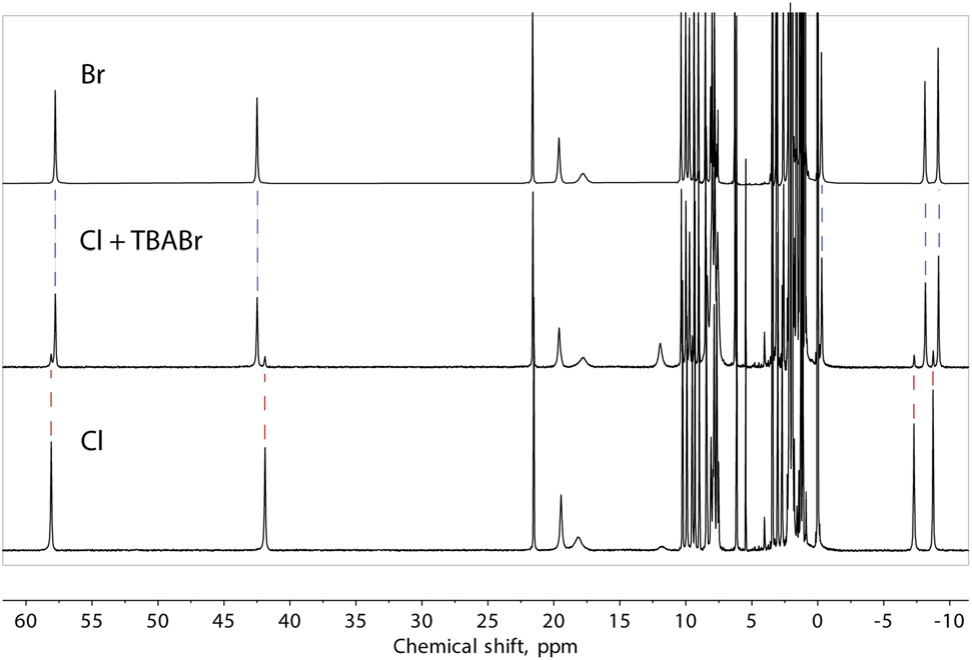
^1^H NMR spectra of ([Fe(L2)_3_]_2_)^4+^, from 1 and (Br@[Fe(L2)_3_]_2_)^3+^, from 2, as well as the spectrum obtained by adding one equivalent of TBA bromide to the solution of 1. Dashed lines are a guide for the eye.

Note that our data do not allow us to differentiate between a truly “empty” ([Fe(L2)_3_]_2_)^4+^ assembly and the species where the cavity is occupied by solvent molecules. However, to obtain evidence of the ability to exchange halide ions with the environment and of the special affinity of the assembly for bromide, we added one equivalent of TBABr to the solution of compound 1 (presumably containing the “empty” ([Fe(L2)_3_]_2_)^4+^ assembly) and observed the immediate formation of the (Br@[Fe(L2)_3_]_2_)^3+^ species (Fig. 8). On the other hand, the addition of an extra source of Cl^−^ to solution of 1 did not induce any changes in the spectrum, confirming the preference of the dimeric assembly ([Fe(L2)_3_]_2_)^4+^ for larger halide ions, especially bromide.

DFT calculations were performed to corroborate the drive of the [Fe(L2)_3_]^2+^ helical complexes to form dimers (details in the ESI†). Thus, the process of eqn (1) in acetonitrile was calculated to liberate 183.1 kcal mol^−1^.

1 2([Fe(L2)_3_])^2+^ + Br^−^ → (Br@[Fe(L2)_3_]_2_)^3+^

The formation of an empty dimer is also favorable, with at least 114.8 kcal mol^−1^ of liberated energy. For comparison, the values calculated for the analogous processes with the shorter ligand L1 are 168.9 and 106.6 kcal mol^−1^, respectively.

The formation of a dinuclear triple stranded helicate was simulated with the process in eqn (2).

2 ([Fe(L)_3_])^2+^ + FeBr_2_ → (Br@[Fe_2_(L)_3_])^3+^ + Br^−^

The calculated liberated energies are 49.6 and 69.6 kcal mol^−1^ for L2 and L1, respectively.

The above results are consistent with the observed exclusive formation of (X@[Fe(L)_3_]_2_)^3+^ dimer assemblies with L2 while both, the dimer and the triple stranded helicate can be isolated, depending on the reaction conditions, for L1.^27,37^

## Conclusions

The engineering of supramolecular interactions allows selection of a self-recognition process of dimerization over one of helicate-cage formation. Thus, the potential formation of a [Fe_2_L_3_]^4+^ helicate is suppressed in favour of a ([Fe(L)_3_]_2_)^4+^ dimer by enhancing the main intermolecular interaction holding it together by virtue of a fused benzene ring at each end of the ligand L. This is realized in the solid state with the crystallization of pure salts of high-spin (X@[Fe(L2)_3_]_2_)^3+^ cationic assemblies, where both components trap an X^−^ anion in between them; Cl^−^ (1), Br^−^ (2) or I^−^ (3). The persistence of the dimers upon dissolution can be established with no ambiguity by paramagnetic NMR spectroscopy. This technique unveiled that the dimer holds Br^−^ strongly in solution, releases Cl^−^ very fast upon dissolution while not dissociating itself and slowly releases I^−^ in between its both components but maintaining the dimer structure.

The ability to tune the molecular recognition in this category of compounds opens an avenue to engineering multifunctional systems by choosing the properties of the components to be assembled.

## Data availability

Crystallographic data are deposited as cif files in the CCDC.

## Author contributions

AR produced some coordination compounds. LAB coordinated all the synthetic work (organic and inorganic) and characterization, conducted some of the NMR experiments and wrote part of the paper. RD produced some of the corrdiantion compounds. OR collected all SCXRD data, solved them and refined them. DYA conducted the variabke temperature NMR experiments.YN contributed to refining some SCXRD data. VN coordianted the NMR experiments, collected some of the NMR spectra and interpreted them, and wrote part of the paper. SJT is the beam line scienteist at 12.2.1 station of ALS and contributied to the SCXRD data collection. JR conducted the DFT calculations. GA coordinated the entire project and wrote most of the paper.

## Conflicts of interest

There are no conflicts to declare.

## Supplementary Material

SC-015-D4SC01365D-s001

SC-015-D4SC01365D-s002
