# Supplementary material for: Engineered π⋯π interactions favour supramolecular dimers X@[FeL3]2 (X = Cl, Br, I): solid state and solution structure
Source: Chem Sci. 2024 May 28;15(24):9047–53. doi: 10.1039/d4sc01365d (PMC11186344; doi:10.1039/d4sc01365d)
Supplement: SC-015-D4SC01365D-s001 [file SC-015-D4SC01365D-s001.pdf]

Electronic Supplementary Material (ESI) for Chemical Science.
This journal is © The Royal Society of Chemistry 2024

# Engineered π···π interactions favour supramolecular dimer X@[$FeL_3$]$_2$ (X=Cl, Br, I): solid state and solution structure

SUPPORTING INFORMATION

## 1. Synthesis

**1,3-bis-(1-(quinolin-2-yl)propane-1,3-dione)-benzene (L'2).** A solution of $^t$BuOK (16mmol) in dry THF (16ml) was added dropwise and with stirring to a solution of 1,3-diacetophenone (0.65g, 4mmol) in dry THF (100ml) under $N_2$. Upon further stirring for 15 min, the solution changed to light-yellow colour. Methyl quinoline-2-carboxylate (1.5g, 8mmols)[1] was then added and the solution was brought to reflux overnight. During this process the mixture turned dark yellow. THF was then evaporated under low pressure, yielding a reddish-brown colour that was rinsed with water and then suspended in a biphasic solution of water (200ml) and diethyl ether (100ml). 37% HCl was added until pH=2 was reached and the mixture was stirred for a few minutes, verifying that the pH remained at 2-3. After this time, the formation of an abundant white precipitate was observed. The latter was filtered under vacuum and 1.49 g of white solid (79.2%) were obtained.

$^1$H NMR (400 MHz, Chloroform-d) δ 16.48 (s, 2H), 8.84 (s, 1H), 8.41 – 8.27 (m, 8H), 7.98 (s, 2H), 7.95 – 7.89 (m, 2H), 7.85 – 7.78 (m, 2H), 7.74 – 7.61 (m, 3H). 2D $^1$H COSY spectrum is shown in Fig. S13.

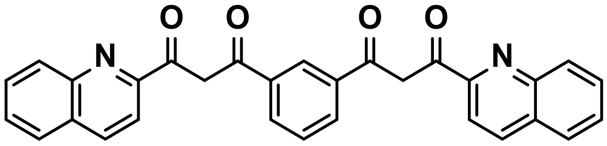


**Figure S1.** Structure of L'2.

**1,3-bis-(3-(quinolin-2-yl)-1H-pyrazol-5-yl)-benzene (L2).** To a yellow solution of L'2 (1.5 g, 3.2 mmol) in methanol (150 mL) was added dropwise monohydrate hydrazine 64% (2.4 mL, 32 mmol). The solution turned red and the mixture was refluxed overnight and then dried at low pressure. The resulting solid was rinsed with water, filtered under vacuum, and washed twice with methanol to obtain 1.223 g (83.2%) of L2 as a white solid. $^1$H NMR (400 MHz, DMSO-$d_6$) δ 13.74 (s, 2H), 8.46 (d, J = 4.0 Hz, 2H), 8.44 (s, 1H), 8.17 (d, J = 8.6 Hz, 2H), 8.06 (d, J = 8.4 Hz, 2H), 7.99 (t, J = 8.5, 1.4 Hz, 2H), 7.89 (t, J = 7.6, 1.7 Hz, 2H), 7.79 (m, J = 8.4, 6.8, 1.4 Hz, 2H), 7.65 (s, 2H), 7.62 – 7.53 (m, 3H). Elemental analysis: Calculated (found) for $C_{30}H_{20}N_6\cdot1.5H_2O$: C: 73.30 (73.03); H: 4.72 (4.31); N: 17.10 (16.98). Mass Spectometry (MALDI-TOF) *m/z* = 465.2, ([L2+H$^+$]$^+$).

**Salts of (X@[Fe(L2)$_3$]$_2$)$^{3+}$ (X$^-$= Cl$^-$, 1; Br$^-$, 2; I$^-$, 3)**

[1] Patent US 2015/0315199. Hoveyda, H. R.; R. M. O.; F. G. L.; D. v. *Novel NK-3 Receptor Selective Antagonist Compunds, Pharmaceutical Composition and Methods for Use in NK-3 Receptor Mediated Disorder*; **2015**.

A suspension of L2 (40.0 mg, 0.086 mmol) in acetone (10 mL) was added dropwise to a solution of $FeX_2$ (0.029 mmol; $X^-$ = $Cl^-$, 3.7 mg, **1**; $Br^-$, 6.3 mg, **2**; $I^-$, 9.0 mg, **3**) in acetone (5 mL). A yellowish cloudy solution was obtained, which was stirred for 40 minutes. Then, $^{n}Bu_4NPF_6$ (33.4 mg, 0.086 mmols) was added in small portions and the resulting yellow slightly cloudy mixture was stirred for a further 10 minutes and then filtered with a nylon membrane. The filtrate was exposed to vapors of diethyl ether yielding crystalline needles after ten days. Yields were in the order of 15-25%. The formulae consistent with SCXRD and elemental analysis were $(Cl@[Fe(L2)_3]_2)(PF_6)_3$ (**1**), $(Br@[Fe(L2)_3]_2)(PF_6)_3$ (**2**) and $(I@[Fe(L2)_3]_2)(I_3)_{0.39}(PF_6)_{2.61}$ (**3**).

Elemental analysis calculated (found) for $Cl@[Fe(L2)_3]_2(PF_6)_3\cdot 3C_3H_6O$, **1**$\cdot 3C_3H_6O$: C, 64.06 (64.47); H, 3.93 (4.05); N, 14.23 (13.97). $Br@[Fe(L2)_3]_2(PF_6)_3\cdot 8H_2O\cdot 3C_3H_6O$, **2**$\cdot 8H_2O\cdot 3C_3H_6O$: C, 60.81 (60.55); H, 4.06 (3.88); N, 13.72 (13.30). $I@[Fe(L2)_3]_2(I_3)_{0.39}(PF_6)_{2.61}\cdot 3H_2O$, **3**$\cdot 3H_2O$: C, 59.94 (60.63); H, 3.52 (3.87); N, 13.98 (13.83).

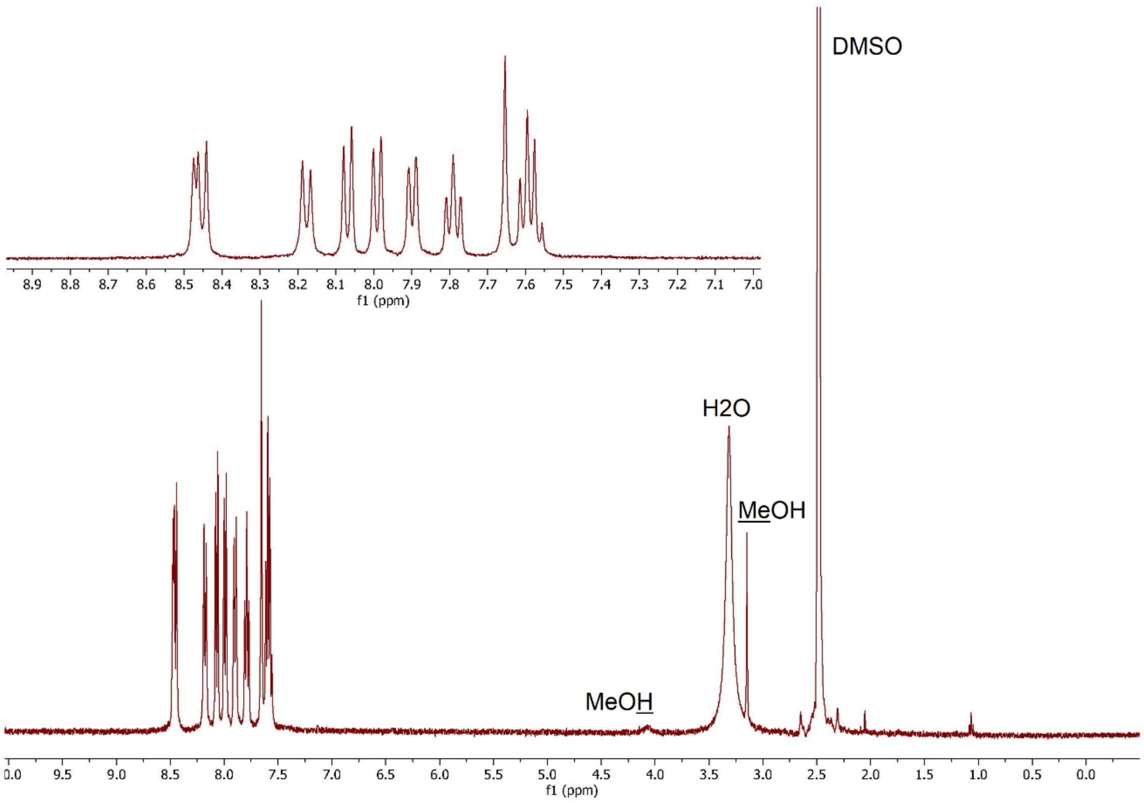


**Figura S2.** 400 HMz $^1H$ NMR spectrum of ligand L2, with an expansion of the aromatic region at the inset. Peaks from various solvents labelled.

## 2. Physical characterization

**Elemental Analysis.** C, H, N analyses were performed by using a Thermo EA Flash 2000 (Thermo Scientific) analyser at the Centres Científics i Tecnològics from the Universitat de Barcelona (CCiT-UB).

**Infrared Spectroscopy.** IR spectra were recorded as KBr pellet samples on a Nicolet 5700 FTIR spectrometer.

**Magnetometry.** Variable-temperature magnetic measurements were performed using a Quantum Design SQUID evercool MXL7 magnetometer at the "Unitat de Mesures Magnètiques" of the Universitat de Barcelona. The diamagnetic contributions to the susceptibility were corrected using Pascal's constant tables. Further corrections of the sample holders contribution, determined empirically, were also applied. Variable temperature *dc* data were collected with an applied field of 5000 Oe, in settle mode.

## 3. Single-crystal X-ray diffraction (SCXRD)

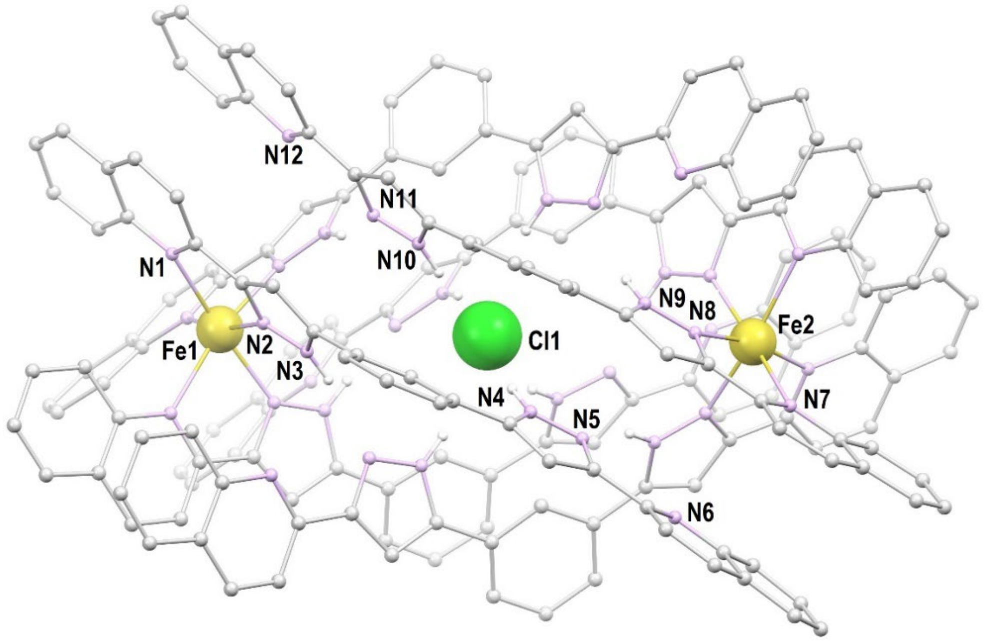


**Figure S3.** Molecular representation of the $(Cl@[Fe(L2)_3]_2)^{3+}$ assembly of **1**. Unique heteroatoms are labelled. Grey balls are C, white balls are H (only H atoms from N−H groups are shown).

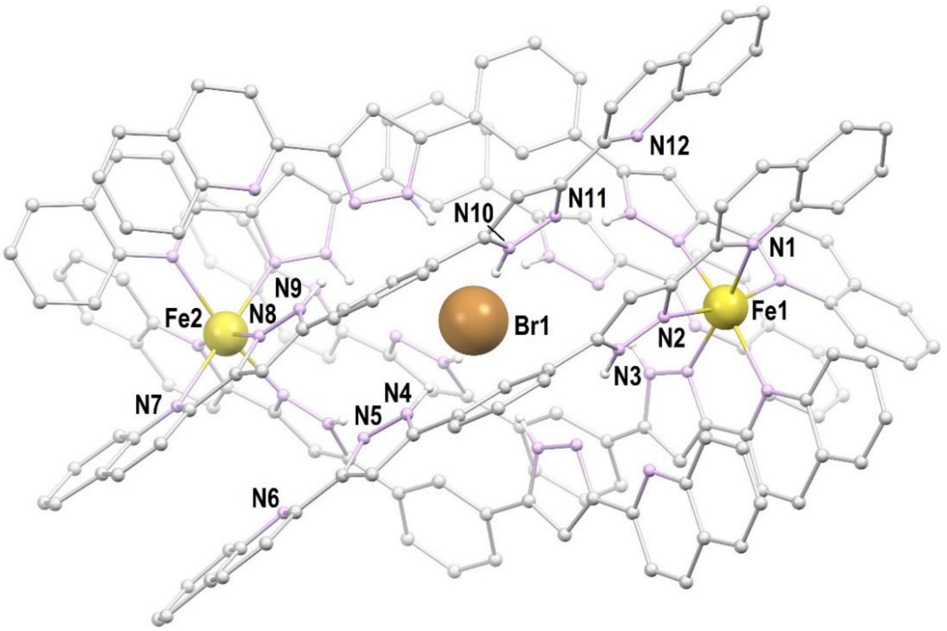


**Figure S4.** Molecular representation of the $(Br@[Fe(L2)_3]_2)^{3+}$ assembly of **2**. Unique heteroatoms are labelled. Grey balls are C, white balls are H (only H atoms from N−H groups are shown).

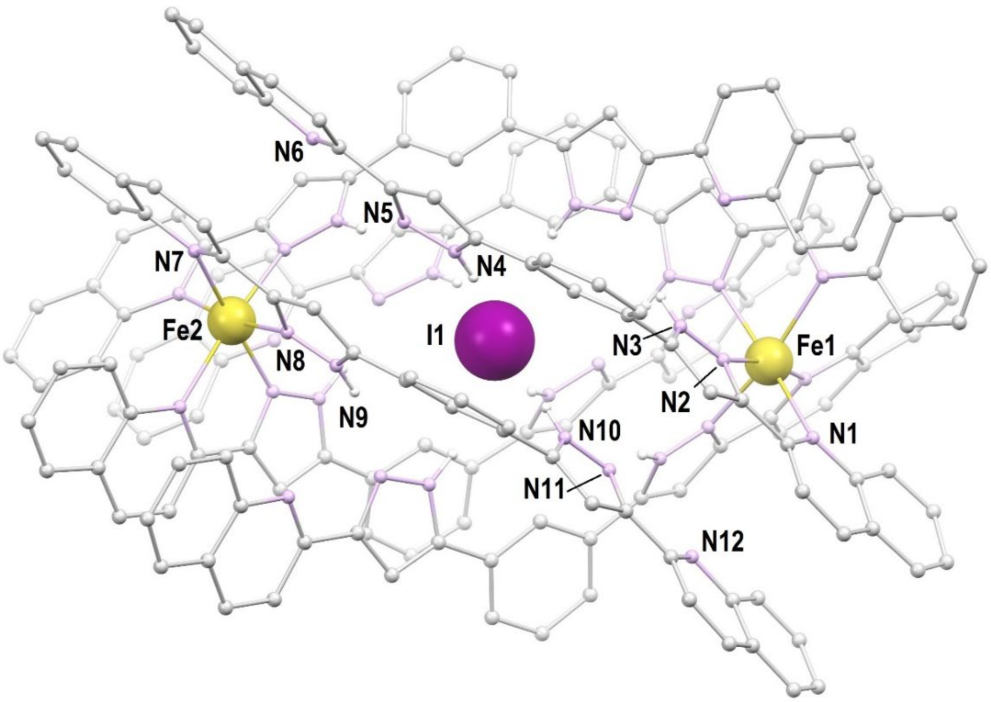


**Figure S5.** Molecular representation of the $(I@[Fe(L2)_3]_2)^{3+}$ assembly of **3**. Unique heteroatoms are labelled. Grey balls are C, white balls are H (only H atoms from N−H groups are shown).

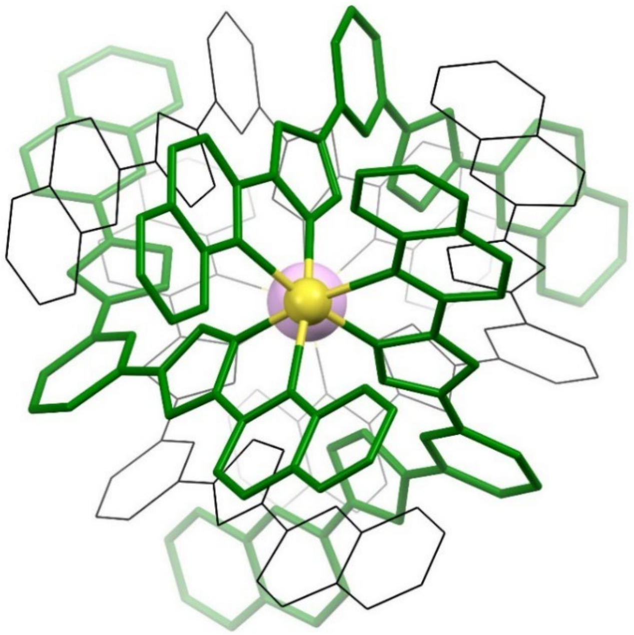

**Figure S6.** Representation of the $(X@[Fe(L2)_3]_2)^{3+}$ supramolecular cations of compounds **1**, **2** and **3** (X = Cl, **1**; Br, **2**; I, **3**), down the axis containing X and the Fe centres. X is purple, Fe is yellow and the ligands for each complex are thick green and thin black sticks, respectively. H atoms are not shown.

## Experimental SCXRD

Data for compounds **1** and **2** were collected at 100 K at Beamline 12.2.1 of the Advanced Light Source (Berkeley, USA), on a Bruker D8 diffractometer equipped with a PHOTON II detector and using silicon (111) monochromated synchrotron radiation ($\lambda$ = 0.7288 Å). Data for compound **3** were acquired at 100 K on the BL13-XALOC beamline[2] of the ALBA synchrotron ($\lambda$ = 0.72932 Å). Data reduction and absorption corrections for **1**, and **2** were performed with respectively SAINT and SADABS.[3] Data reduction for compounds **3** was done with autoproc package[4] and XDS.[5] All structures were solved by intrinsic phasing with SHELXT[6] and refined by full-matrix least-squares on $F^2$ with SHELXL.[7] Free refinement of the position of hydrogens atoms of the pyrazole N-H group did not converge, probably due to the too faint corresponding electron density peak present in difference Fourier maps. These hydrogens were therefore refined with their thermal parameter 1.5 times that of their carrier N atom and a distance restraint at 1.0 angstrom, based on the structure of free pyrazole reported using both X-ray and neutron diffraction.[8] The rest of hydrogens were placed geometrically and refined with a riding model. In the structures of **1**, **2** and **3**, lattice solvent molecules were too diffuse/disordered to be modelled satisfactorily so they have been treated as a diffuse contribution to the overall scattering without specific atom positions by PLATON/SQUEEZE.[9]

All details can be found in CCDC 2279862-2279863-2279864 (**1**-**2**-**3**), which contain the supplementary crystallographic data for this paper. These data can be obtained free of charge from The Cambridge Crystallographic Data Center via https://summary.ccdc.cam.ac.uk/structure-summary-form. Crystallographic and refinement parameters are summarized in Table S1**,** Table S2 provides Fe–N bond lengths while Tables S3 and S4, details of hydrogen bonds and stacking interactions, respectively.

---

[2] J. Juanhuix, F. Gil-Ortiz, G. Cuní, C. Colldelram, J. Nicolás, J. Lidón, E. Boter, C. Ruget, S. Ferrer and J. Benach, *J. Synchrotron Radiat.*, 2014, **21** , 679-689
[3] a) G. M. Sheldrick, *SAINT and SADABS*, 2012, Bruker AXS Inc., Madison, Wisconsin, USA; b) L. Krause, R. Herbst-Irmer, G. M. Sheldrick, D. Stalke, *J. Appl. Cryst.*, 2015, **48**, 3-10.
[4] C. Vonrhein, C. Flensburg, P. Keller, A. Sharff, O. Smart, W. Paciorek, T. Womack, and G. Bricogne, *Acta Cryst. D,* 2011, **67**, 293-302.
[5] W. Kabsch, *Acta Cryst. D* 2010, **66**, 125-132.
[6] G. M. Sheldrick, *Acta Cryst. A* 2015, **71**, 3-8.
[7] G. M. Sheldrick, *Acta Cryst. C* 2015, **71**, 3-8.
[8] F. Krebs Larsen, M. S. Lehmann, I: sotofte and S. E. Rasmussen, Acta Chem. Scan., 1970, 24, 3248-3258
[9] A. L. Spek, *Acta Cryst. C* 2015, **71**, 9-18.

**Table S1.** Crystallographic and refinement parameters for the structures of compounds **1**, **2** and **3**.

| Compound | **1**·4acetone | **2**·4acetone | **3**·$H_2O$·5acetone |
|---|---|---|---|
| Formula | $C_{192}H_{144}ClF_{18}Fe_2N_{36}O_4P_3$ | $C_{192}H_{144}BrF_{18}Fe_2N_{36}O_4P_3$ | $C_{195}H_{152}F_{15.66}Fe_2I_{2.17}N_{36}O_6P_{2.61}$ |
| FW (g mol$^{-1}$) | 3601.48 | 3645.94 | 3878.72 |
| Wavelength (Å) | 0.7288 | 0.7288 | 0.72932 |
| $T$ (K) | 100(2) | 100(2) | 100(2) |
| Crystal system | cubic | cubic | cubic |
| Space group | $P2_13$ | $P2_13$ | $P2_13$ |
| $a$ (Å) | 26.5187(5) | 26.5373(7) | 26.532(3) |
| $b$ (Å) | 26.5187(5) | 26.5373(7) | 26.532(3) |
| $c$ (Å) | 26.5187(5) | 26.5373(7) | 26.532(3) |
| $\alpha$ (°) | 90 | 90 | 90 |
| $\beta$ (°) | 90 | 90 | 90 |
| $\gamma$ (°) | 90 | 90 | 90 |
| $V$ (Å$^3$) | 18649.0(11) | 18688.3(15) | 18677(6) |
| $Z$ | 4 | 4 | 4 |
| $\rho_{calcd}$ (g cm$^{-3}$) | 1.283 | 1.296 | 1.379 |
| $\mu$ (mm$^{-1}$) | 0.294 | 0.505 | 0.703 |
| Reflections | 9289 | 10649 | 18241 |
| $R_{int}$ | 0.0175 | 0.0590 | 0.0305 |
| Restraints | 154 | 244 | 136 |
| Parameters | 797 | 797 | 765 |
| Flack parameter | 0.015(3) | 0.035(5) | 0.031(3) |
| $S$ | 1.066 | 1.034 | 1.067 |
| $R_1$ [$I$>2$\sigma$($I$)] | 0.0505 | 0.0742 | 0.0542 |
| w$R_2$ [$I$>2$\sigma$($I$)] | 0.1473 | 0.2072 | 0.1595 |
| $R_1$ [all data] | 0.0527 | 0.0890 | 0.0546 |
| w$R_2$ [all data] | 0.1505 | 0.2221 | 0.1602 |
| Largest peak / hole ($e$ Å$^3$) | 0.492 / –0.236 | 1.432 / –1.109 | 1.026 / –0.542 |

**Table S2.** M-N bond lengths, M···M and M···X separations (in Å) in the structures of compounds **1**, **2** and **3**.

| | **1** | **2** | **3** |
|---|---|---|---|
| Fe1–N1 | 2.319(4) | 2.316(7) | 2.313(3) |
| Fe1–N2 | 2.144(4) | 2.149(6) | 2.154(3) |
| <Fe1–N> | 2.231(8) | 2.232(13) | 2.233(6) |
| Fe2–N7 | 2.311(4) | 2.317(8) | 2.313(4) |
| Fe2–N8 | 2.132(5) | 2.145(7) | 2.138(4) |
| <Fe2–N> | 2.221(8) | 2.231(15) | 2.225(8) |
| Fe1···X | 6.091(2) | 6.088(2) | 6.101(1) |
| Fe2···X | 6.056(2) | 5.985(2) | 5.856(1) |
| Fe1···Fe2 | 12.147(2) | 12.073(2) | 11.957(2) |

**Table S3.** Hydrogen bonds involved in the supramolecular X@[Fe(L2)$_3$]$_2$ assemblies in the structures of compounds **1**, **2** and **3**.

| | D–H···A | D–H (Å) | H···A (Å) | D···A (Å) | D–H···A (º) |
|---|---|---|---|---|---|
| **1** | N4–H4B···Cl1 | 0.97(3) | 2.49(4) | 3.376(4) | 153(5) |
| | N10–H10A···Cl1 | 0.99(3) | 2.49(4) | 3.341(3) | 145(4) |
| | N3–H3B···N11 | 0.95(3) | 1.96(3) | 2.889(5) | 166(5) |
| | N9–H9A···N5 | 0.96(3) | 2.00(3) | 2.931(6) | 164(6) |
| **2** | N4–H4B···Br1 | 1.00(3) | 2.45(4) | 3.422(6) | 161(8) |
| | N10–H10A···Br1 | 0.99(3) | 2.44(4) | 3.406(6) | 165(7) |
| | N3–H3B···N11 | 0.98(3) | 1.91(3) | 2.882(9) | 168(8) |
| | N9–H9A···N5 | 0.98(3) | 1.96(4) | 2.93(1) | 168(9) |
| **3** | N4–H4B···I1 | 0.95(3) | 2.61(3) | 3.518(3) | 159(5) |
| | N10–H10A···I1 | 0.99(3) | 2.65(4) | 3.526(3) | 148(4) |
| | N3–H3B···N11 | 1.00(3) | 1.93(3) | 2.905(4) | 164(5) |
| | N9–H9A···N5 | 0.96(3) | 2.02(3) | 2.981(5) | 176(6) |

**Table S4.** Inter-centroid (Å) and interplane angles (°)* of seven unique parallel-displaced stacking interactions in the supramolecular X@[Fe(L2)$_3$]$_2$ assemblies.

| | | a | b | c | d | e | f | g |
|---|---|---|---|---|---|---|---|---|
| **1** | Cg···Cg (Å) | 3.918 | 3.671 | 3.547 | 3.776 | 3.567 | 3.610 | 3.801 |
| | interplane angle (º) | 8.38 | 7.26 | 2.49 | 5.28 | 3.81 | 4.51 | 5.75 |
| **2** | Cg···Cg (Å) | 3.943 | 3.679 | 3.562 | 3.790 | 3.574 | 3.600 | 3.814 |
| | interplane angle (º) | 8.96 | 7.51 | 2.31 | 4.26 | 4.20 | 4.55 | 5.76 |
| **3** | Cg···Cg (Å) | 3.924 | 3.663 | 3.575 | 3.841 | 3.620 | 3.585 | 3.827 |
| | interplane angle (º) | 8.58 | 7.95 | 2.64 | 3.65 | 4.41 | 4.88 | 7.25 |

a: C1C2C3C4C5C6···C55C56C57C58C59C60; b: N1C6C7C8C9C10···N12C52C53C54C55C60
c: N2N3C10C11C12···N10N11C49C50C51; d: C13C14C15C16C17C18···C43C44C45C46C47C48
e: N4N5C19C20C21···N8N9C40C41C42; f: N6C22C23C24C25C30···N7C36C37C38C39CC31
g: C25C26C27C28C29C30···C31C32C33C34C35C36

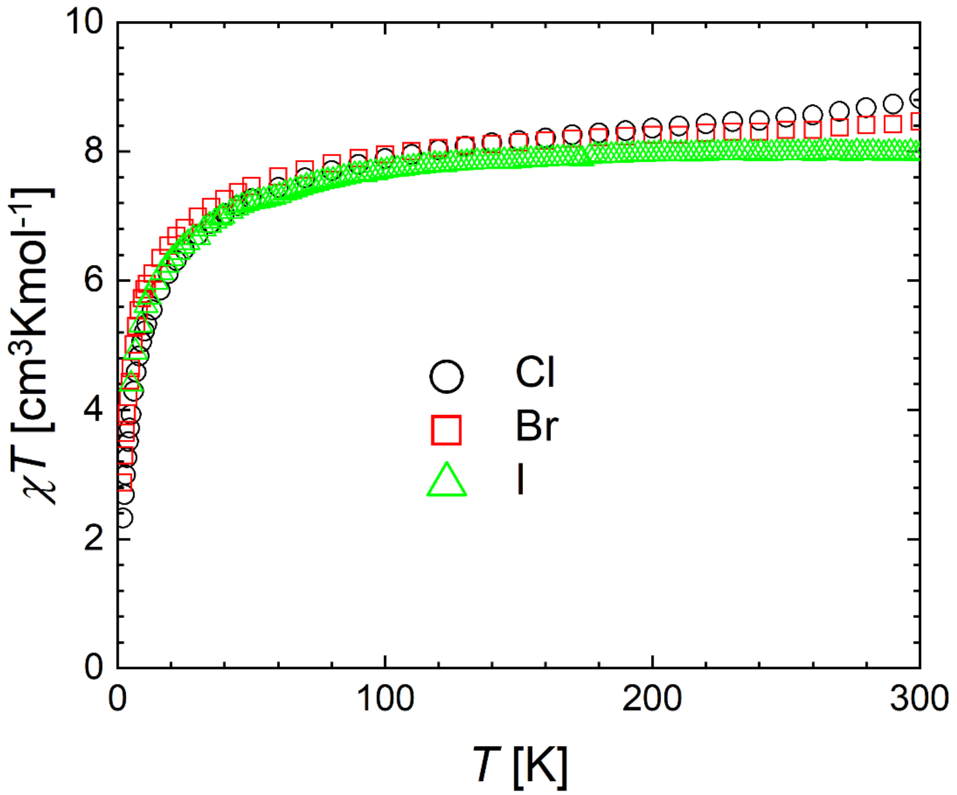


**Figure S7.** Plots of $\chi T$ *vs* $T$ for compounds **1** (Cl), **2** (Br) and **3** (I), in the 2 (Cl and Br) or 4 (I) to 300 K temperature range. The value of the plateau is above that expected for two isotropic $Fe^{2+}$ centers in the HS state ($S$ = 2) with $g$ = 2 and no spin-orbit coupling contributions (6 $cm^3Kmol^{-1}$). The observed data may be subject to small errors ascribed to the attributed molar mass (dependent on the exact amount of solvation molecules or moisture). The drop at lower temperatures is expected for this ion, subject to zero field splitting effects and the consequent failure of the Curie law in this regime.

## 4. Paramagnetic NMR

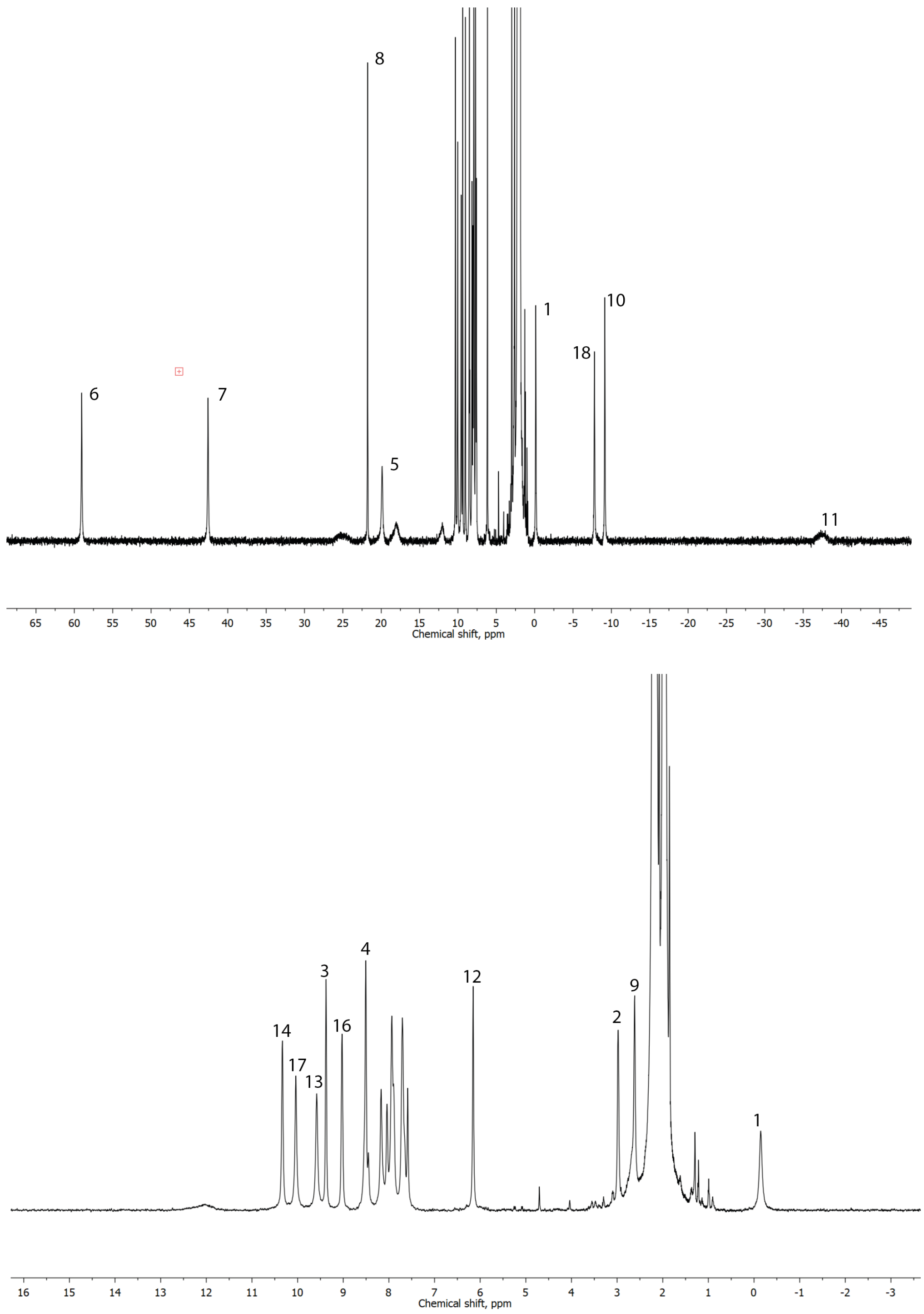


**Figure S8.** Paramagnetic (top) and diamagnetic (bottom) regions of the $^{1}H$ NMR spectra of compound **1**; X=Cl ($CD_3CN$, 295 K, 600 MHz).

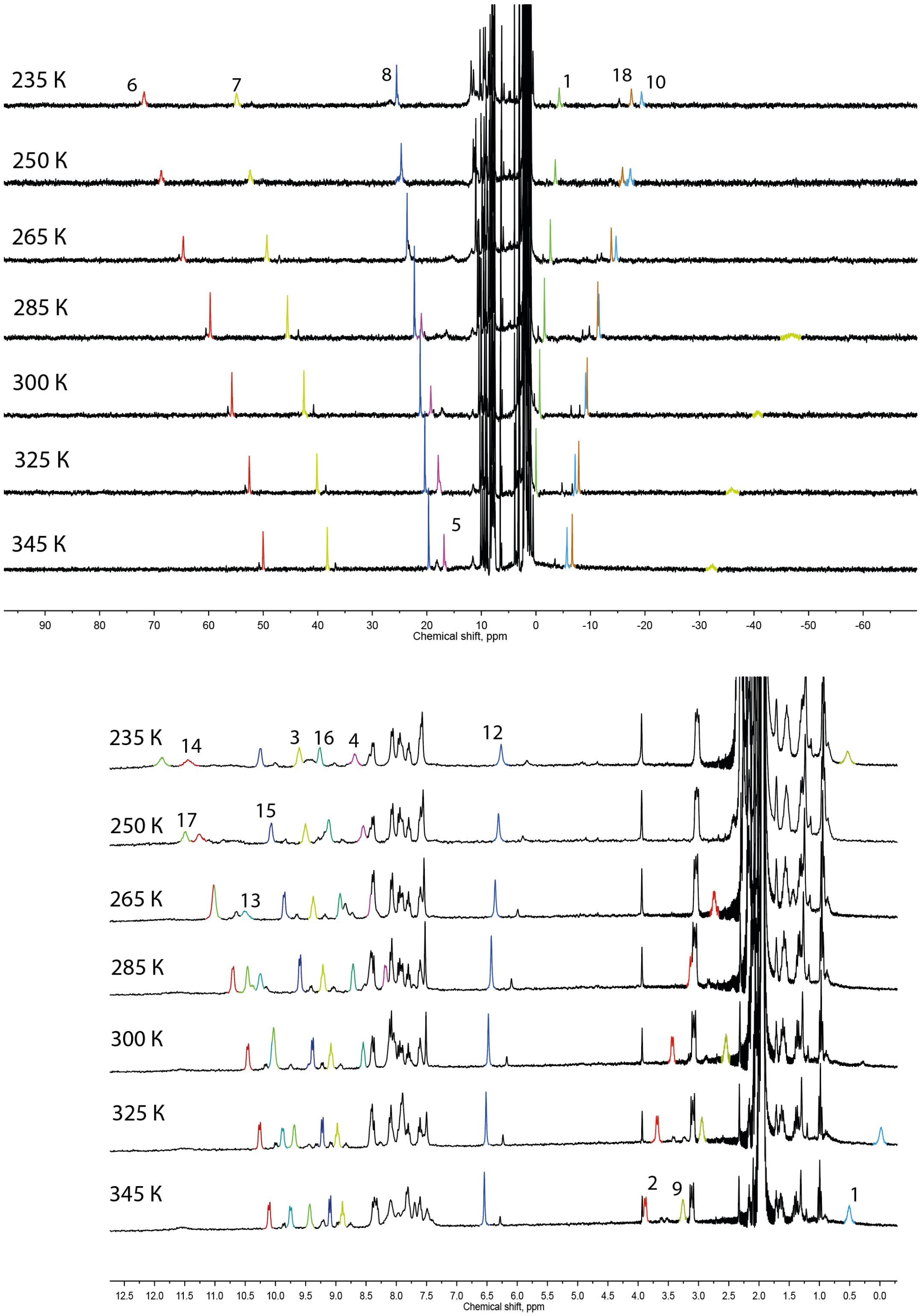


**Figure S9.** Paramagnetic (top) and diamagnetic (bottom) regions of the $^1$H NMR spectra of compound **3**; X= I ($CD_3CN$, 300 MHz).

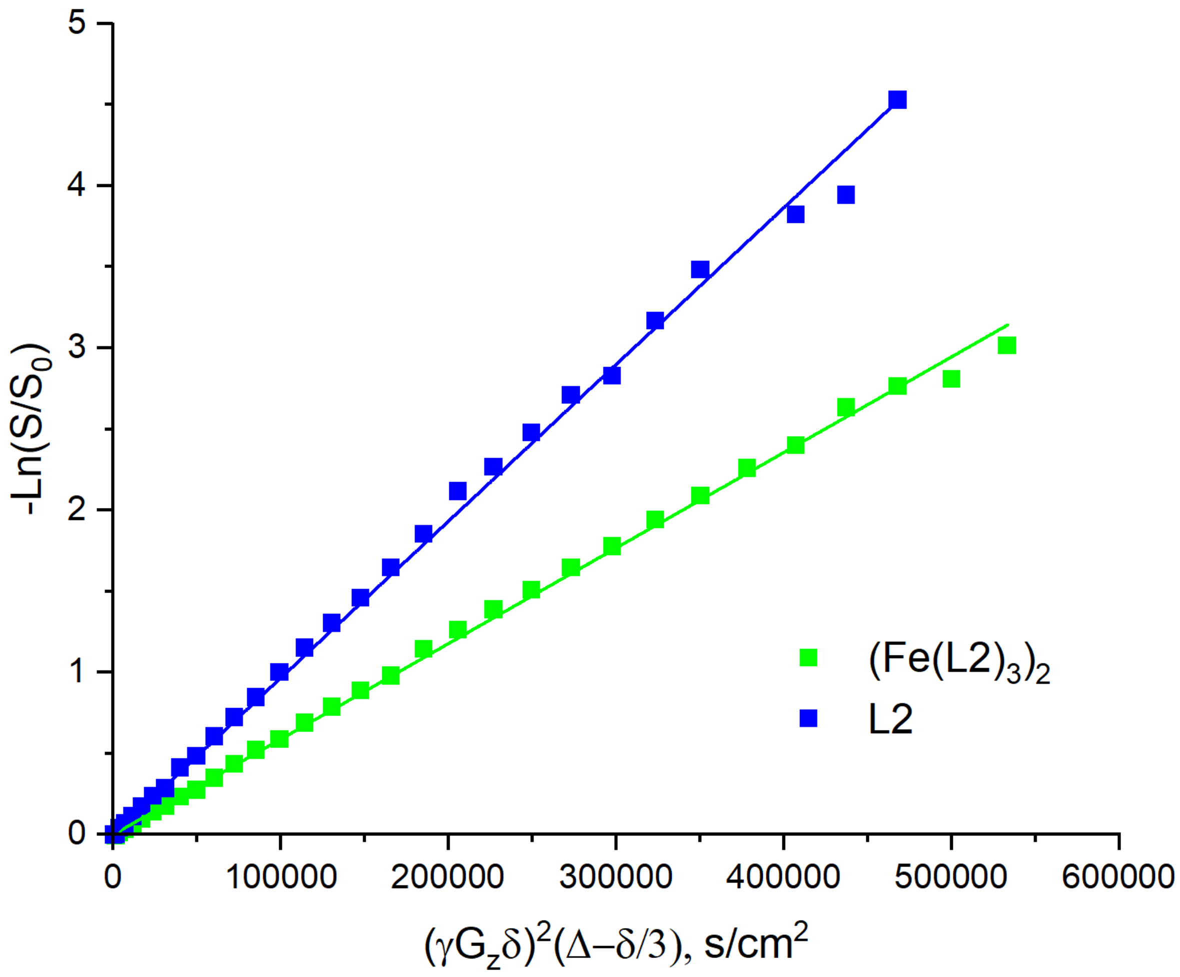


**Figure S10.** $^{1}H$ NMR DOSY data for compound **2** (X=Br). To minimize the effect of the paramagnetic relaxation, the diffusion coefficients were calculated using the signals of the $(Br@[Fe(L2)_3]_2)^{3+}$ moiety in the range 8.5 – 10.5 ppm and of free ligand in the range 7.4 – 8.6 ppm, followed by averaging the $S/S_0$ intensity ratio. The diffusion coefficient was determined as slope of a tangent line in coordinates –ln($S/S_0$) *vs* $(\gamma G_z\delta)^2(\Delta-\delta/3)$. The calculated diffusion coefficients were D = 9.66 $cm^2/s$ for free ligand L2 and D = 5.89 $cm^2/s$ for the species $(Br@[Fe(L2)_3]_2)^{3+}$.

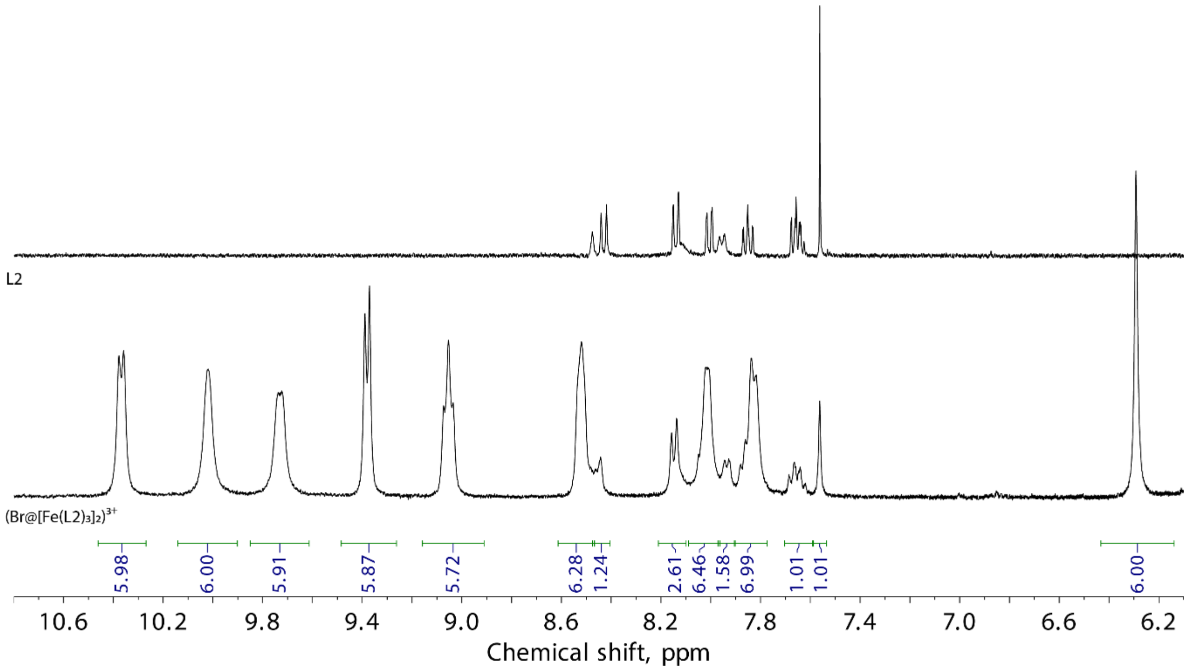


**Figure S11.** Comparison of aromatic diamagnetic region of $^{1}H$ spectrum of compound **2,** X = Br (bottom) with the one for the free ligand L2 (top). Conditions: $CD_3CN$, 400 MHz, room temperature. The sample of compound **2** was obtained by preliminary washing the single crystals of **2** with tetrahydrofuran (a good solvent for free ligand that does not dissolve the complex **2**) with further drying and dissolution in $CD_3CN$.

The ratio of the concentrations of the compound **2** and free ligand **L2** in solution was estimated based on the comparison of integrals of pyrazole protons of complex ($\delta$ = 6.29 ppm, I = 6.00) and the ligand ($\delta$ = 7.56 ppm, I = 1.01). As the ligand has two-fold symmetry, absent in the complex, and each complex molecule has six ligands, the resulting 6-to-1 ratio of integrals corresponds to ratio of 2:1 of molar concentration of **2** to **L2** in solution. In other words, only one out of each thirteen **L2** molecules is free in solution, and the rest are present as the part of the complex.

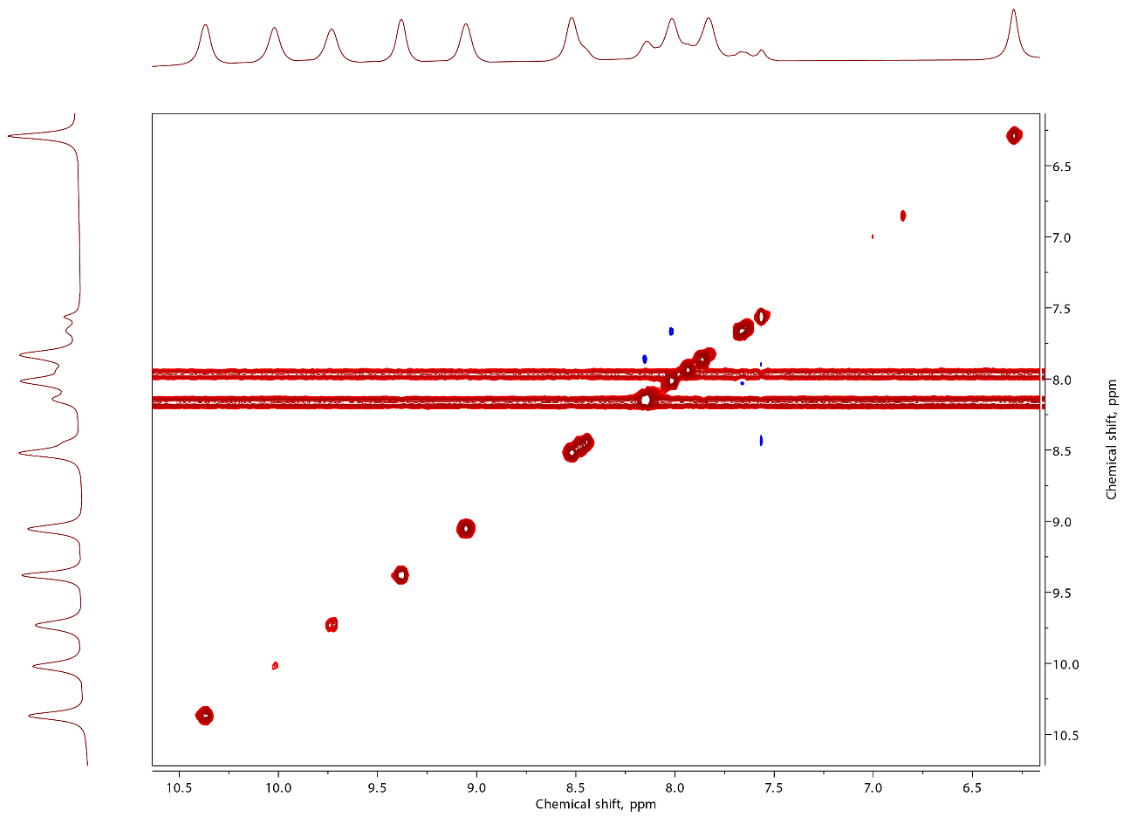


**Figure S12.** $^{1}$H 2D EXSY spectrum of compound **2**; X=Br ($CD_3CN$, 400 MHz) at room temperature. The weak blue cross peaks are NOE-type dipolar cross-peaks between the signals of the protons of the free ligand. There are no exchange cross-peaks with the same sign as diagonal peaks, implying the absence of the exchange phenomena in the NMR timescale.

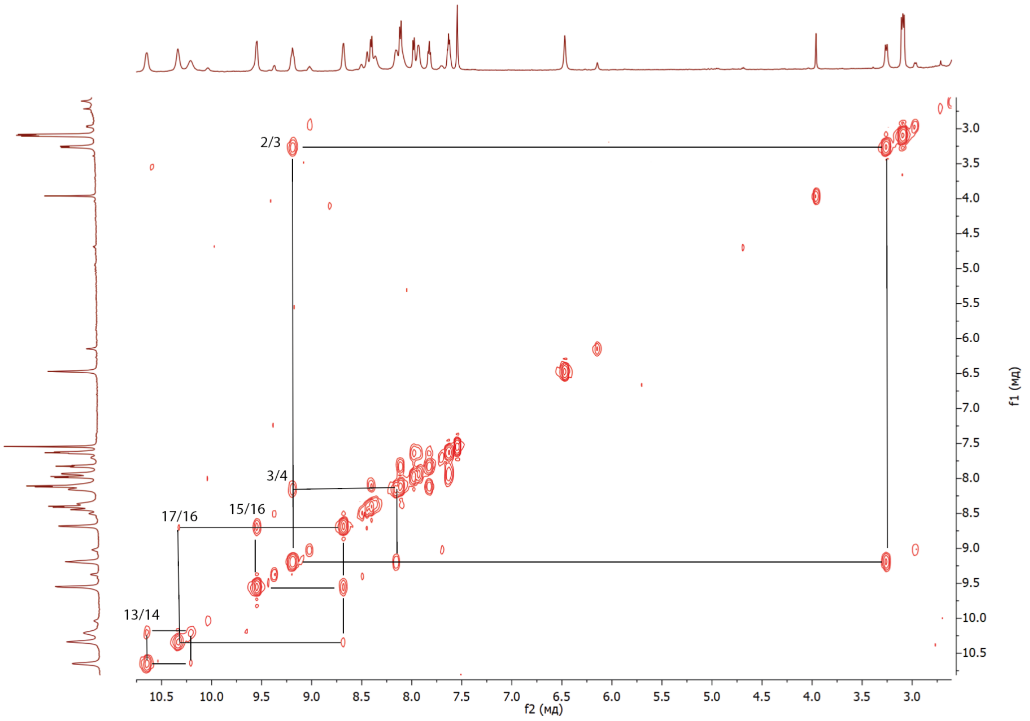


**Figure S13.** COSY spectrum of compound **3**; X= I ($CD_3CN$, 600 MHz) at room temperature.

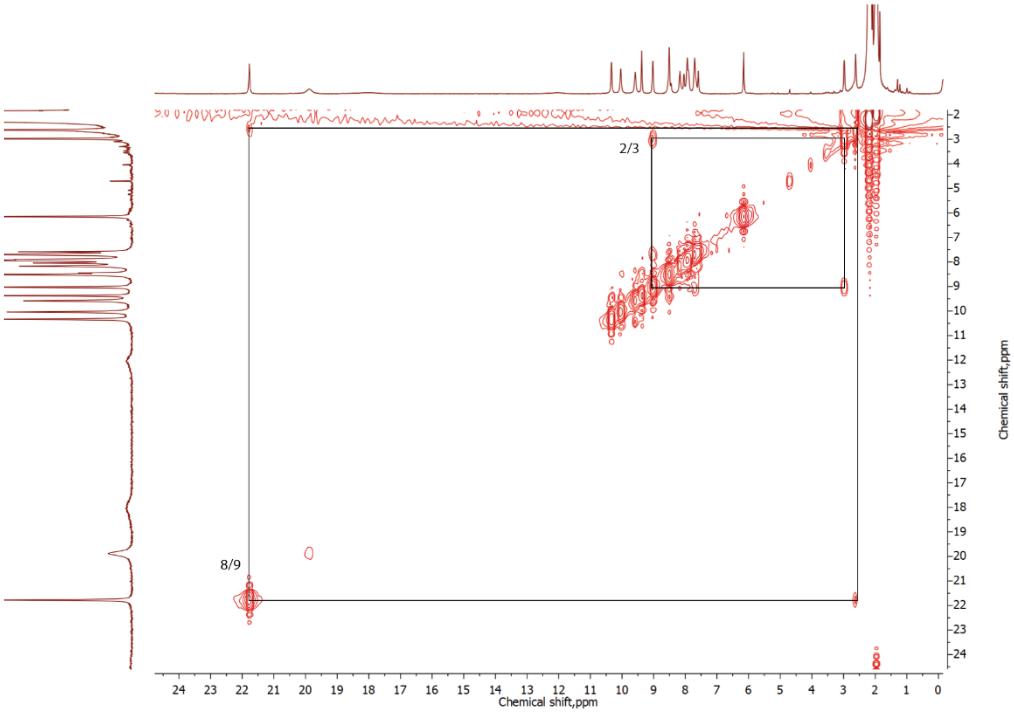


**Figure S14.** COSY spectrum of compound **1**; X= Cl ($CD_3CN$, 300 MHz) at room temperature.

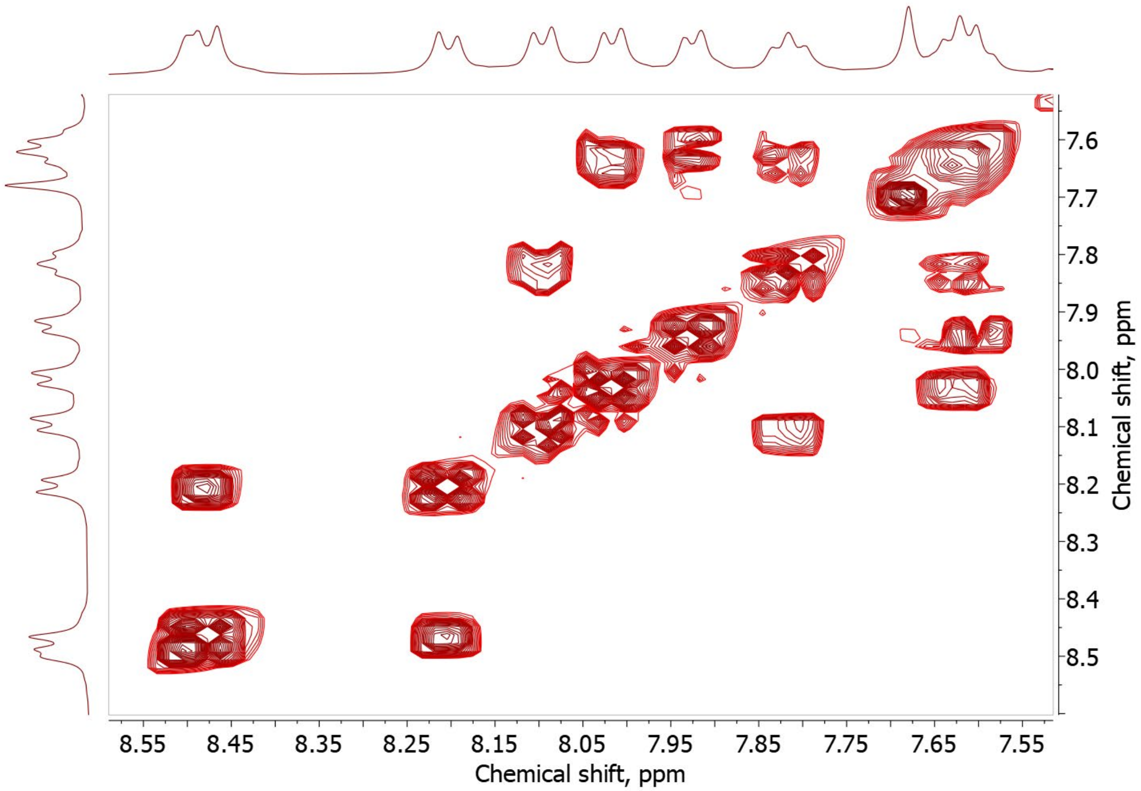


**Figure S15.** $^1$H NMR COSY spectrum for L2 ligand (DMSO, 400 MHz).

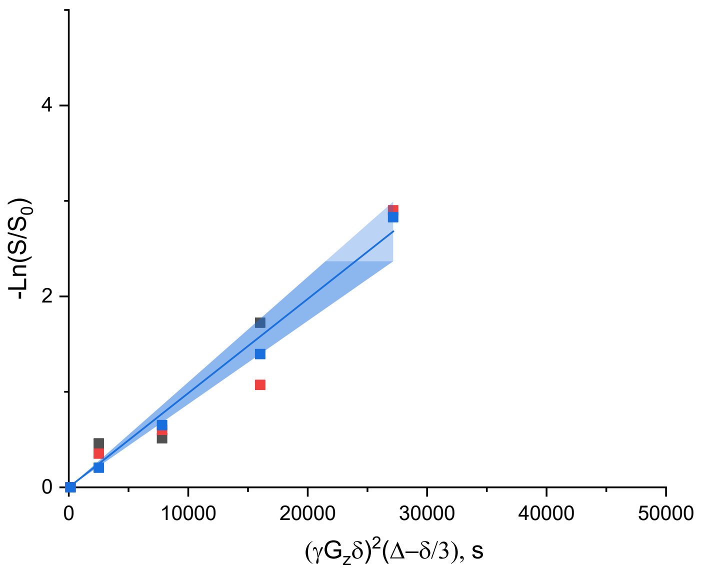


$[Fe(L2)_3]_2^{4+}$

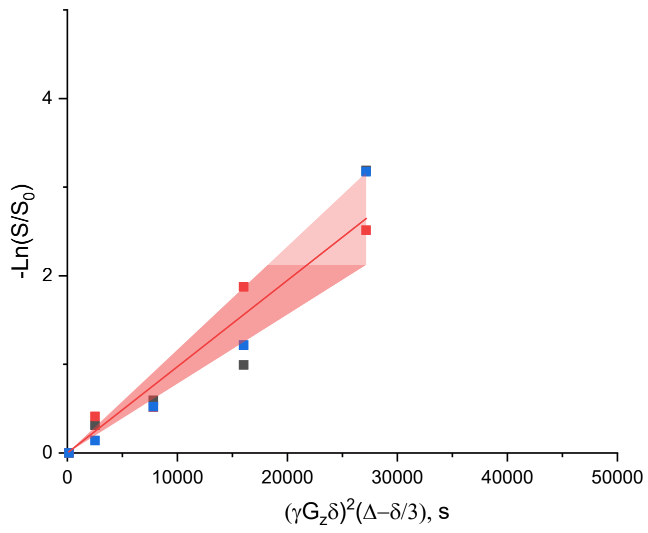


$(I@[Fe(L2)_3]_2)^{3+}$

**Figure S16.** $^1$H NMR DOSY data for compound **3** (X=I). The colored symbols correspond to three spectral regions at 6.3 (black), 9.3 (red) and 10.5 ppm, where the signals of $(I@[Fe(L2)_3]_2)^{3+}$ and $([Fe(L2)_3]_2)^{4+}$ assemblies are well-resolved. The diffusion coefficient was determined as a slope of a tangent line in coordinates –ln($S/S_0$) *vs* $(\gamma G_z\delta)^2(\Delta-\delta/3)$. The ratio of the diffusion coefficients for species $(I@[Fe(L2)_3]_2)^{3+}$ and $[Fe(L2)_3]_2^{4+}$ was 1.03.

| Proton | $([Fe(L2)_3]_2)^{3+}$ | $(Br@[Fe(L2)_3]_2)^{3+}$ | $(I@[Fe(L2)_3]_2)^{3+}$ |
|---|---|---|---|
| 1 | -0.1 | -0.5 | -1.3 |
| 2 | 3.0 | 3.1 | 3.2 |
| 3 | 9.0 | 9.0 | 9.2 |
| 4 | - | 7.8 | 8.1 |
| 5 | 19.8 | 20.0 | 20.4 |
| 6 | 59.0 | 59.0 | 58.3 |
| 7 | 42.5 | 43.3 | 44.5 |
| 8 | 21.7 | 21.9 | 21.9 |
| 9 | 2.6 | 2.6 | 2.2 |
| 10 | -7.8 | -8.8 | -10.7 |
| 11 | -37.0 | -39.9 | -38.8 |
| 12 | 6.1 | 6.2 | 6.4 |
| 13 | 9.5 | 9.8 | 10.2 |
| 14 | 10.3 | 10.4 | 10.6 |
| 15 | 9.3 | 9.4 | 9.5 |
| 16 | 8.4 | 8.5 | 8.7 |
| 17 | 10 | 10.1 | 10.3 |
| 18 | -9.1 | -9.7 | -10.7 |

**Table S5.** Assignment of the paramagnetic NMR spectra at room temperature for compounds **1**, **2** and **3**.

## $^{1}$H NMR experimental data

$^{1}$H and $^{1}$H-$^{1}$H COSY NMR spectra were recorded from solutions in acetonitrile-$d_3$ with a Bruker Avance 600 and Bruker Avance 300 FT-spectrometer (600.2 MHz and 300.1 MHz $^{1}$H frequency) using the residual signals of the solvent ($^{1}$H 1.94 ppm) as internal standards. To calibrate the temperature within the temperature range 190 – 300 K, a Bruker standard temperature calibration sample (4% of MeOH in methanol-$d_4$) was used. Above 300 K, the temperature was calibrated using a known dependence of the chemical shifts of pure ethylene glycol. The DOSY spectrum was obtained using a standard bipolar pulse sequence (ledbpgp2s) and the following parameters: acquisition time 3 s, relaxation delay 1 s, diffusion time 50 ms, gradient pulse length 1.25 ms. Diffusion coefficients were determined as the slope of tangent lines in coordinates $-\ln(S/S_0)$ *vs* $(\gamma G_z \delta)^2(\Delta-\delta/3)$.

## Computational Details

The energy gain upon formation of the dimers ($[Fe(L1)_3]_2)^{4+}$ and ($[Fe(L2)_3]_2)^{4+}$ was evaluated after fully optimizing their geometry and that of the corresponding monomers. These structural optimizations were carried out using density functional theory (DFT) based calculations employing the PBE[10] exchange-correlation functional and a def2-SVP[11] basis set using the Gaussian 16 code.[12] We have opted for using the PBE functional, instead of a hybrid functional (e.g. B3LYP), as a compromise between computational cost and accuracy. It is worth mentioning that PBE has already been successfully used to model systems with FeII ions in a high-spin state.[13] Spin-unrestricted calculations were done when dealing with all the species because the high-spin of the Fe(II) ions were considered. In all the calculations, the DFT-D3(BJ) semi-empirical dispersion potential introduced by Grimme[14] was added to the conventional Kohn–Sham energy for a proper description of the van der Waals interactions. Calculations in acetonitrile were performed using the polarizable continuum model.[15]

---

[10] J.P. Perdew, K. Burke, M. Ernzerhof, Phys. Rev. Lett. 1996, 77, 3865–3868.

[11] F. Weigend, R. Ahlrichs, Phys. Chem. Chem. Phys., 2005, 7, 3297-3305.

[12] Gaussian 16, Revision B.01, M. J. Frisch, G. W. Trucks, H. B. Schlegel, G. E. Scuseria, M. A. Robb, J. R. Cheeseman, G. Scalmani, V. Barone, G. A. Petersson, H. Nakatsuji, X. Li, M. Caricato, A. V. Marenich, J. Bloino, B. G. Janesko, R. Gomperts, B. Mennucci, H. P. Hratchian, J. V. Ortiz, A. F. Izmaylov, J. L. Sonnenberg, D. Williams-Young, F. Ding, F. Lipparini, F. Egidi, J. Goings, B. Peng, A. Petrone, T. Henderson, D. Ranasinghe, V. G. Zakrzewski, J. Gao, N. Rega, G. Zheng, W. Liang, M. Hada, M. Ehara, K. Toyota, R. Fukuda, J. Hasegawa, M. Ishida, T. Nakajima, Y. Honda, O. Kitao, H. Nakai, T. Vreven, K. Throssell, J. A. Montgomery, Jr., J. E. Peralta, F. Ogliaro, M. J. Bearpark, J. J. Heyd, E. N. Brothers, K. N. Kudin, V. N. Staroverov, T. A. Keith, R. Kobayashi, J. Normand, K. Raghavachari, A. P. Rendell, J. C. Burant, S. S. Iyengar, J. Tomasi, M. Cossi, J. M. Millam, M. Klene, C. Adamo, R. Cammi, J. W. Ochterski, R. L. Martin, K. Morokuma, O. Farkas, J. B. Foresman, and D. J. Fox, Gaussian, Inc., Wallingford CT, 2016.

[13] Ribas-Arino *et al.* *J. Am. Chem. Soc.* **2006**, 128, 9497.

[14] S. Grimme, S. Ehrlich and L. Goerigk, J. Comp. Chem. 2011, 32, 1456-65.

[15] J. Tomasi, B. Mennucci, and R. Cammi, Chem. Rev., 2005, 105, 2999-3093.
